# *Sphallerocarpus gracilis* Polyphenols Alleviate DSS-Induced Colitis by Remodeling Gut Microbiota and Inhibiting the AGE-RAGE/HMGB1 Pathway

**DOI:** 10.3390/antiox15091130

**Published:** 2026-09-07

**Authors:** Xuanjun Wang, Jun Zhang, Saizhen Guo, Wenbo Zhang, Ziyan Yang, Dengyou Nie, Zemin Xiang, Yongkai Xi

**Affiliations:** 1 College of Science, Yunnan Agricultural University, Kunming 650051, China; xjwang@cxtc.edu.cn (X.W.); 2024210074@stu.ynau.edu.cn (J.Z.); 15608808921@163.com (S.G.); 15912059075@163.com (W.Z.); 18487681845@163.com (D.N.); 2 Key Laboratory of Development and Utilization of Food and Medicinal Resources, Ministry of Education, Yunnan Agricultural University, Kunming 650051, China; 13577244560@163.com; 3 College of Agronomy, Chuxiong Normal University, Chuxiong 675099, China; 4 College of Food Science and Technology, Yunnan Agricultural University, Kunming 650051, China; 5 The Key Laboratory of Agricultural Microbiome of Yunnan Province, Yunnan Agricultural University, Kunming 650051, China

**Keywords:** *Sphallerocarpus gracilis* polyphenols, ulcerative colitis, AGE-RAGE signaling pathway, *Akkermansia muciniphila*, gut microecological modulation

## Abstract

As a traditional Tibetan medicine and health food, the fleshy roots of *Sphallerocarpus gracilis* were adopted to extract polyphenol powder(PP) in this research. Using a DSS-induced colitis mouse model combined with multi-omics analysis and experimental validation, we explored the protective effects and molecular mechanisms of PP against intestinal inflammation. PP mitigated colitis-related symptoms in a dose-dependent manner, inhibited pro-inflammatory cytokines and dose-dependently reversed elevated IL-10 levels to BC group. Multi-omics data revealed that PP intervention was associated with gut microbiota remodeling, enrichment of *Akkermansia muciniphila*, restoration of bacterial–fungal homeostasis, and improvement of tryptophan and biotin metabolism. By suppressing HMGB1 and RAGE expression, PP blocked the AGE-RAGE-mediated inflammatory cascade. In addition, PP maintained intestinal mucosal integrity through goblet cell protection, MDA reduction, and the regulation of BAX/Bcl-2, MMP3 and MMP9. Overall, PP relieves UC by regulating gut microecology, host metabolism and the AGE-RAGE pathway, which supports the translational potential of natural polyphenols for inflammatory bowel disease treatment as a gut microecological modulator.

## 1. Introduction

Ulcerative colitis (UC) is a chronic, relapsing intestinal disease and a subtype of inflammatory bowel disease (IBD). It has been reported in major developed countries worldwide, and both its incidence and hospitalization rates show an upward trend [1]. The precise pathogenesis of UC remains unclear. Notably, a research group isolated a bacterial strain designated Macrophage-Toxic Bacteria from the fecal samples of affected individuals, and found that it secretes aerolysin, which exerts pathogenic effects. They accordingly proposed a therapeutic strategy targeting bacterial virulence factors [2].

The inflammation associated with UC typically occurs in the intestinal mucosal layer. Studies have shown that intestinal inflammatory responses are highly site-specific. On the one hand, epithelial cells in different segments possess developmentally preset, distinct immune sensing capacities [3]; on the other hand, chronic inflammation further reshapes epithelial cell identity by inducing the emergence of metaplastic cells that are normally absent from the region, thereby altering local tissue architecture and the immune microenvironment [4]. The intestinal epithelial barrier is not a passive physical partition; disruption of its integrity can directly contribute to the initiation and perpetuation of IBD by increasing the translocation of luminal antigens [5]. Accordingly, UC is frequently regarded as an epithelial barrier disease [6,7]. Nevertheless, the initial pathogenic factors that trigger epithelial barrier dysfunction remain unclear.

Unfortunately, no curative treatment currently exists for this disease. Clinically used medications such as corticosteroids, aminosalicylates, and immunosuppressants are associated with drawbacks including low oral bioavailability, high cost, and various adverse effects [8]. If conventional pharmacological therapies fail, the risk of progression from colonic inflammation to colorectal cancer increases significantly, and patients may require colectomy.

Therefore, compounds obtained from natural products have emerged as a promising direction for UC treatment [9]. Studies have shown that targeted nano-delivery systems designed based on bioactive constituents of traditional Chinese medicine can exert therapeutic effects through multiple pathways, including anti-inflammatory actions, barrier repair, and modulation of the gut microbiota [10,11]. Similarly, traditional Chinese medicine formulas [12], fermented products [13], and natural foods [14,15] have also been demonstrated to ameliorate colitis through comprehensive mechanisms involving the regulation of inflammation, oxidative stress, the intestinal barrier, and the microbiome. Among the natural constituents of plants, polyphenols are a class of compounds widely distributed in nature [16], possessing antiviral [17], anti-ovarian aging [18], antibacterial [19], and digestion-regulating [20] properties. A large body of epidemiological evidence indicates that the intake of certain amounts of plant polyphenols plays a positive role in disease prevention and treatment [21,22,23].

The pathogenesis of UC involves intestinal barrier disruption, inflammatory dysregulation, and microbiota imbalance, against which polyphenols exert multi-targeted effects. These compounds interrupt pro-inflammatory cytokine cascades by suppressing the TLR4/NF-κB/NLRP3 pathway [24] and PI3K-AKT signaling [25], restore mucosal integrity by upregulating tight junction proteins and mucins, and attenuate oxidative stress through Nrf2 activation [26,27]. Additionally, polyphenols remodel the gut microbiota by enriching short-chain fatty acid-producing beneficial bacteria while inhibiting opportunistic pathogens [28]. Genetic evidence from Mendelian randomization has further confirmed a negative association between polyphenol intake and UC risk [29], while dietary polyphenols derived from foods such as apples and jujubes have been shown to alleviate UC-associated behavioral disorders [30,31]. Multiple studies have also demonstrated that flavonoids possess anti-inflammatory, antioxidant, mucosa-healing, and intestinal immune homeostasis-maintaining bioactivities, exhibiting favorable efficacy in alleviating UC and holding broad application prospects [32,33].

*Sphallerocarpus gracilis* (Besser ex Trevir.) Koso-Pol (*S. gracilis*) is a perennial plant and the sole species within the genus *Sphallerocarpus*, typically growing at high altitudes. Its primary medicinal part is the dried fleshy rhizome, which is cylindrical in shape with a yellowish-brown surface. In many regions of China, *S. gracilis* is commonly used as a traditional health food [34]. According to ancient records, this plant has been used to treat ulcers, tetanus, and other diseases, and is listed as a traditional Tibetan medicine [35]. *S. gracilis* can also serve as a principal ingredient in medicinal formulas, such as “Liuwei Migouqin San” and “Wugeng San,” which are indicated for joint swelling, abdominal distension, picky eating, and anorexia. However, its mechanisms of action remain poorly understood. The crude polysaccharides extracted from *S. gracilis* have been applied in dairy product processing [36], can modulate the gut microbiota and metabolic functions in mice [37], and exhibit potent antioxidant activity [38]. Meanwhile, polyphenols extracted from the stems and leaves of *S. gracilis* have demonstrated significant hepatoprotective effects and protection against DNA damage [39,40,41]. Polyphenols extracted from *S. gracilis* seeds also show protective effects against DNA damage [42]. These findings suggest that the major bioactive constituents of *S. gracilis* may be its polyphenols and polysaccharides. However, these findings are inconsistent with the traditional medicinal uses of *S. gracilis*. We hypothesized that root polyphenols are the major active components responsible for its intestinal protective effects, and accordingly designed this study to evaluate their intervention in a DSS-induced colitis model.

In this study, we employed a DSS-induced murine colitis model to evaluate the protective effects of *Sphallerocarpus gracilis* root polyphenols extract (PP) on the intestine. The gut microbiota in the colonic contents was characterized. By integrating untargeted metabolomics and network pharmacology, we predicted pathways and potential targets associated with UC pathogenesis. The mechanism of action of PP in colitis was further validated experimentally. This study elucidates the molecular mechanisms underlying the dual effects of these PP, providing a theoretical basis for exploring natural products that promote intestinal health and prevent colitis.

## 2. Materials and Methods

### 2.1. Biological Materials

*S. gracilis* samples were collected in August 2024 at the Shandan Army Horse Ranch in Gansu Province (Coordinates: 38°18′15″ N, 101°03′42″ E; Altitude: 2913 m). Immediately after collection, the samples were frozen on dry ice and subsequently stored at −80 °C for long-term preservation.

After identification by Professor Xia Fan of the Kunming Institute of Botany, Chinese Academy of Sciences, and verification against The World Flora Online (http://www.worldfloraonline.org/taxon/wfo-0000434795. Accessed on: 27 May 2025), the fleshy roots were confirmed as those of *S. gracilis*. The plant samples are deposited in the specimen repository of The Agricultural Chemistry Research Center at Yunnan Agricultural University (Voucher number: YNAU-ACRC-20250823013).

### 2.2. Extraction of Sphallerocarpus gracilis Polyphenols

Following the method of Ma et al. [39,40] with slight modifications, polyphenols were extracted from *S. gracilis*. Fresh *S. gracilis* was washed, dried at 35 °C, pulverized, and passed through a 60-mesh sieve. The *S. gracilis* powder was mixed with 75% ethanol at a solid-to-liquid ratio of 1:35 (g/mL) and subjected to ultrasound-assisted extraction at 40 kHz and 70 °C for 30 min. After centrifugation at 4000 rpm, the supernatant was collected; this extraction was repeated three times. The combined supernatants were concentrated by rotary evaporation under reduced pressure to obtain the *S. gracilis* extract. The extract was deproteinized using the Sevag method. The deproteinized extract was then purified using X-5 macroporous resin. The pH of the extract solution was adjusted to 2.0, and the solution was loaded onto the resin column at a concentration of 2.0 mg/g. Elution was performed with 70% ethanol at a flow rate of 1.5 mL/min, and the first 400 mL of eluate was collected. The eluate was concentrated under reduced pressure and lyophilized to obtain *S. gracilis* polyphenol powder (PP).

### 2.3. Animal Experiments

All Five-week-old male C57BL/6 mice weighing 20–30 g were purchased from SPF Biotechnology Co., Ltd. (Beijing, China). (Enterprise Animal License No.: SCXK (JING) 2024-0001). All animal procedures were performed in strict accordance with the guidelines approved by The life science Ethics Committee of Yunnan Agricultural University and were approved by the committee (IACUC No: APYNAU 202504033, Experimental animal usage License number: SYXK(DIAN)K2021-0002). Mice were housed in a specific-pathogen-free (SPF) facility under a 12 h light/dark cycle at a controlled temperature (22 ± 2 °C) and humidity (50–60%), fed with a regular chow diet (Jiangsu Synergy Bio-Products Co., Ltd., Nanjing, China) and water. All efforts were made to minimize animal suffering, following the ‘3Rs’ principles (Replacement, Reduction, and Refinement) throughout the study.

After a 7-day acclimation period, C57BL/6 mice were divided into four groups (*n* = 7): Blank control (BC, saline), Model control (MC, A 3% (*w*/*v*) DSS solution was prepared in sterile saline [43]), PP-H treatment group (PP high-dose, 80 mg/kg PP-H + DSS), PP-L treatment group (PP Low-dose, 40 mg/kg, PP-L + DSS). To minimize potential cage effects, animals from each treatment group were distributed across at least two cages, and no experimental group was confined to a single cage. DSS solution was freshly prepared at 3% weight/volume in sterile drinking water and filtered through a 0.22 μm filter before use. Mice in the MC, PP-L, and PP-H groups received DSS solution as their sole drinking source ad libitum, and the daily intake of DSS solution was recorded. PP was dissolved in sterile saline and administered daily by oral gavage using a stainless-steel feeding needle at a volume of 100 μL per mouse, at approximately 9:00 AM each day. The BC group received an equal volume of sterile saline by gavage following the same schedule. Body weights were recorded every other day throughout the 8-day experimental period. DSS was administered at 3% concentration based on preliminary experiments and previous literature to induce reproducible colitis, and PP was given via oral gavage to mimic the natural route of dietary polyphenol intake.

A total of 28 mice (7 per group × 4 groups) were used in this experiment. Mice were randomly assigned to the four experimental groups using a computer-generated random number sequence (Excel, RAND function) to ensure unbiased allocation.

Treatments and measurements were performed in a fixed order and were not randomized. However, all procedures were standardized with respect to time of day, handling conditions, and operator technique to minimize variability. Cage location was controlled as described above. The individual mouse was considered as the experimental unit for all phenotypic and molecular analyses.

The sample size (*n* = 7 per group) was determined based on previous studies using the DSS-induced colitis model with similar interventions, which demonstrated sufficient statistical power to detect significant differences in body weight change, DAI score, and colon length among groups [44,45].

The mice in the BC group were gavaged with 100 μL saline; rats in the model were provided with DSS solution in place of sterile drinking water, while those in the PP-H and PP-L treatment groups received daily oral gavage for 8 consecutive days, with body weights recorded every other day.

**Figure 1:**
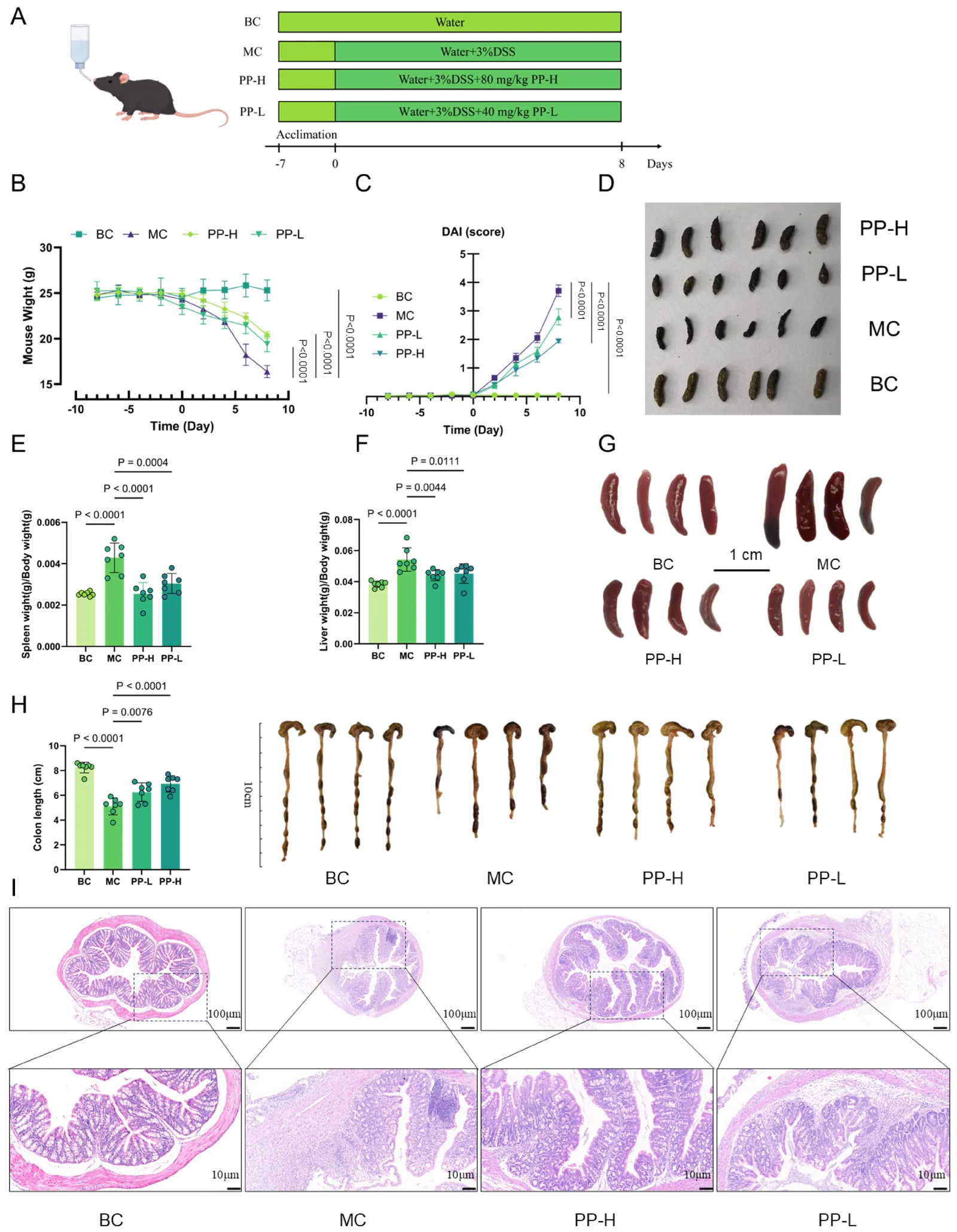
Effect of PP intervention on the murine ulcerative colitis model. (**A**) Experimental design and grouping (**B**) Body weight change (*n* = 7). (**C**) Disease activity index (DAI) scores during the experimental period (*n* = 7). (**D**) Fecal morphology observation during the experimental period. (**E**) Spleen weight/body weight ratio (*n* = 7). (**F**) Liver weight/body weight ratio (*n* = 7). (**G**) Representative images of spleen tissues. (**H**) Colon length measurement and representative images of colon tissues (*n* = 7). (**I**) Representative H&E-stained colon sections. Data are presented as mean ± SD. Data in (**C**,**E**–**G**,**I**) were analyzed by one-way ANOVA.

The in vivo experiments involving PP were conducted using a protocol analogous to that described above. Based on previous literature, a dosage of 40 mg/kg was selected [46]. PP was administered via oral gavage concurrently with DSS exposure. Mice received 2.5% DSS in drinking water for 8 days, after which they were sacrificed. All detailed plans are shown in Figure 1A.

Subsequently, daily treatments were administered until the end of the experiment. The daily intake of sterile water and DSS solution was recorded concurrently.

A priori exclusion criteria were defined as follows: for the BC, PP-L, and PP-H groups, any animal exhibiting > 30% body weight loss relative to initial weight would be excluded from the analysis. This criterion was not applied to the MC group, as substantial body weight loss is an expected pathological feature of the DSS-induced colitis model. During the experiment, no animals in any group met these criteria, and all animals were included in the final analysis.

The primary outcome measure was defined as the disease activity index (DAI), which was used to monitor disease progression and determine the experimental endpoint on day 8. No unexpected adverse events were observed in any group throughout the study. On day 8, fresh fecal samples were collected under aseptic conditions, snap-frozen in liquid nitrogen, and subsequently stored at −80 °C. Blood was then collected from the retro-orbital sinus under anesthesia, and serum was isolated and stored at −80 °C for subsequent experiments. After euthanasia by cervical dislocation, the colons were promptly harvested, washed with PBS, and measured for length. Spleens were excised, weighed, and photographed. Colon tissues were also collected for further analysis: a small portion was used for histological sectioning, while the remainder was frozen at −80 °C for future experiments.

Group allocation was not blinded during the conduct of the experiment, as the DSS solution and PP treatments were visibly different. However, DAI scoring was performed in a blinded manner by two independent observers, as described in Section 2.4. Outcome assessments, including histological evaluation, immunofluorescence quantification, Western blot densitometry, and ELISA, were conducted by investigators blinded to group allocation. Data analysis was performed using coded sample identifiers to ensure unbiased interpretation.

The sample size for each analysis is indicated in the corresponding figure legend, with *n* = 7 for phenotypic and molecular biology assays, *n* = 5 for 16S/ITS sequencing, and *n* = 6 for metabolomics.

For multi-omics and molecular analyses, samples that failed to meet predefined quality control criteria (DNA concentration < 10 ng/μL, protein concentration < 0.5 μg/μL, or tissue weight < 20 mg) were excluded from the corresponding assays, with the specific exclusion details reported in each respective method subsection.

### 2.4. Disease Activity Index (DAI)

The disease activity index (DAI) score assesses disease severity by recording weight loss, stool consistency, and the presence of blood in the stool [47]. DAI scoring was performed in a blinded manner by two independent observers, and any discrepancies were resolved through discussion to reach consensus, thereby minimizing interobserver variability and ensuring the reliability of the assessment. The DAI scoring criteria are listed in Supporting Information Table S2.

### 2.5. H&E and PAS Staining

Following deparaffinization and rehydration, intestinal tissue sections were stained with hematoxylin for 5–10 min to render the nuclei blue-purple. The sections were then differentiated in acid alcohol for a few seconds and blued in ultrapure water for 5–10 min to clearly visualize the blue nuclei. Subsequently, the sections were stained with eosin for 1–3 min to stain the cytoplasm and other components pink. The sections were then dehydrated through a graded ethanol series, cleared in xylene, and mounted with neutral balsam. Images were captured using a Leica DMIL LED microscope (Wetzlar, Germany).

For PAS staining, after deparaffinization and rehydration, the intestinal tissue sections were oxidized with periodic acid, rinsed with distilled water, and then stained with Schiff reagent in the dark until the target structures appeared purplish-red. The sections were subsequently rinsed with sodium bisulfite solution, washed with ultrapure water, and counterstained with hematoxylin. Following differentiation, bluing, dehydration, and clearing, the sections were mounted and imaged using a Leica DMIL LED microscope (Wetzlar, Germany).

### 2.6. Immunofluorescence Technique of Tissue

Following a previously described method [48], mouse colons were collected, flushed, fixed, dehydrated, and embedded in paraffin to prepare 4-μm sections. After permeabilization and blocking, the sections were incubated with antibodies against High mobility group box (HMGB1, 1:250), Epithelial cadherin (E-cadherin, 1:250), and Receptor for advanced glycation end products (RAGE, 1:250) overnight at 4 °C. Following rinses, the sections were incubated with secondary antibodies for 1 h at room temperature in the dark and mounted with DAPI-containing mounting medium. Images were acquired using a Leica upright fluorescence microscope, and the channel colors were adjusted as follows: E-cadherin, red; HMGB1, green; RAGE, yellow; nuclei, blue. Goblet cells were quantified in PAS-stained sections by counting the number of goblet cells per crypt in a blinded manner. No composite histological damage score was applied.

### 2.7. Enzyme-Linked Immunosorbent Assay (ELISA)

Mouse venous blood was collected and centrifuged to obtain the supernatant. The concentrations of AGE, IL-1β, TNF-α, MDA, IL-10, IL-6, and HMGB1 in the serum were analyzed using commercial assay kits (Shanghai DuMa Biotechnology Co., Ltd., Shanghai, China) and a microplate reader, strictly following the manufacturer’s instructions. Goblet cells were quantified in PAS-stained sections by counting the number of goblet cells per crypt in a blinded manner. No composite histological damage score was applied.

### 2.8. Western Blotting

Fresh mouse colonic tissues were collected, rinsed with ice-cold PBS, and homogenized on ice in RIPA lysis buffer (Solarbio, Beijing, China). The homogenate was centrifuged at 12,000 rpm for 15 min at 4 °C, and the supernatant was collected. Protein concentration was determined using a BCA assay kit (Beyotime, Shanghai, China). After adding loading buffer, the samples were heated at 100 °C for 8 min for denaturation. Proteins were separated by SDS-PAGE and wet-transferred onto a pre-activated PVDF membrane. The membrane was blocked with 5% non-fat milk at room temperature for 1 h, and then incubated with primary antibodies overnight at 4 °C. Following washes, the membrane was incubated with secondary antibodies at room temperature for 1 h. Finally, ECL substrate (Biosharp, Beijing, China) was added, and protein bands were detected using a chemiluminescence imaging system (FluorChem E, ProteinSimple Inc., Santa Clara, CA, USA).

### 2.9. 16S rRNA Gene Sequencing and Analysis

Under sterile conditions, the mouse colon was collected, incised longitudinally, and the luminal contents were extruded. The contents were aliquoted, snap-frozen in liquid nitrogen, and stored at −80 °C. Approximately 100 mg of sample was used for total DNA extraction; a blank extraction control was included. DNA quality was assessed, and the concentration was normalized. The V3–V4 region of the 16S rRNA gene was amplified using the primers 338F (ACTCCTACGGGAGGCAGCAG) and 806R (GACTACHVGGGTATCTAATCC). The PCR products were purified, quantified, pooled in equimolar amounts for library construction, and subjected to paired-end sequencing on an Illumina NovaSeq 6000 platform. Data were analyzed using the Myvi Cloud Analysis Platform.

16S rRNA amplicon sequencing was performed on the MGI paired-end platform. Effective sequences ranged from 64,165 to 83,089 per sample (mean 72,054). Quality filtering used fastp 0.23.4 (adapter trimming, removal of reads with ambiguous bases or >40% low-quality bases below Q15, truncation at 4 bp window with mean Q < 20, polyG removal, and discarding merged reads < 150 bp), and paired-end reads were assembled with FLASH 1.2.11 (minimum overlap 10 bp, maximum mismatch 0.1). ASVs were generated using Deblur (QIIME 2 2024.5) instead of OTU clustering. Taxonomy was assigned by Mothur 1.48 against SILVA 138.1 (confidence 0.8–1.0). Samples were rarefied to 64,165 sequences, with rarefaction curves reaching plateaus. LEfSe 1.1.2 used the Kruskal–Wallis test (*p* < 0.05), LDA score threshold of 4, and Benjamini–Hochberg correction for FDR < 0.05.

Six samples from the MC,BC,PP-H group from the 16S rRNA sequencing analysis were excluded due to insufficient DNA quality, with the A260/A280 ratio falling below 1.8, which failed to meet the quality control criteria for library construction. The raw 16S rRNA sequences obtained in this study have been deposited in the National Center for Biotechnology Information (NCBI) database (BioProject:PRJNA1502527).

### 2.10. Bioinformatics Analysis of ITs Gene Sequences

Approximately 100 mg of mouse colonic contents was collected, snap-frozen in liquid nitrogen, and stored at −80 °C. Total DNA was extracted using a fecal genomic DNA extraction kit, and DNA quality was assessed by agarose gel electrophoresis and a microvolume spectrophotometer. Using the extracted total DNA as a template, the ITS1-5.8S region of the fungal ribosomal RNA gene was amplified with the primers ITS1-5F (5′-GGAAGTAAAAGTCGTAACAAGG-3′) and ITS2 (5′-GCTGCGTTCTTCATCGATGC-3′). The PCR program consisted of an initial denaturation at 95 °C for 5 min, followed by 35 cycles of denaturation at 95 °C for 30 s, annealing at 52 °C for 30 s, and extension at 72 °C for 1 min, with a final extension at 72 °C for 7 min. The PCR products were purified with magnetic beads, quantified by PCR, and pooled in equimolar amounts for library construction. Paired-end sequencing was performed on an Illumina NovaSeq 6000 platform, and data were analyzed using the Myvi Cloud Analysis Platform (Myvi Metabolism, Wuhan, China).

For fungal ITS sequencing (also MGI paired-end), effective sequences ranged 64,877–151,582 (mean 98,526). Quality control and assembly were identical to 16S. ASVs were generated with Deblur, annotated by Mothur against UNITE 10 (confidence 0.8–1.0). Rarefaction to 64,877 sequences yielded plateaued curves. LEfSe parameters were the same as for 16S (Kruskal–Wallis *p* < 0.05, LDA score ≥ 4, FDR < 0.05).

Six samples from the MC, BC, PP-H groups were excluded from the ITS sequencing analysis because the PCR amplification failed to generate sufficient product for library preparation, as determined by agarose gel electrophoresis. The raw ITS data obtained in this study have been deposited in the National Center for Biotechnology Information (NCBI) database (BioProject:PRJNA1502527).

### 2.11. Non-Targeted Metabolomics Analysis

Mouse intestinal tissues were snap-frozen in liquid nitrogen and stored at −80 °C. After thawing on ice, 20 mg of tissue was weighed and homogenized with a steel bead using a ball mill, followed by centrifugation for 30 s. A methanolic aqueous internal standard extraction solution was added, and the mixture was vortexed at 1500 rpm for 5 min and left to stand on ice for 15 min. After centrifugation at 12,000 rpm for 10 min at 4 °C, the supernatant was collected and left to stand at −20 °C for 30 min. Following another centrifugation, 200 μL of the supernatant was transferred to an injection vial. Analysis was performed using a Thermo Vanquish system coupled to a Q Exactive HF-X mass spectrometer (Thermo Fisher Scientific, Bremen, Germany) with a Waters ACQUITY Premier HSS T3 column. For the PP sample, 50 mg of powder was weighed and mixed with a pre-cooled 70% methanolic aqueous internal standard extraction solution. The mixture was vortexed for 30 s every 30 min for a total of six cycles, centrifuged for 3 min, and the supernatant was filtered through a 0.22 μm filter membrane and stored in an injection vial for UPLC-MS/MS analysis. Detailed parameters are provided in Supporting Information Tables S3–S6.

One sample from the BC group, one sample from the MC group, and three samples from the PP-H group were excluded from the metabolomics analysis because their total ion chromatogram (TIC) exhibited significant deviation from the quality control (QC) samples, indicating possible sample degradation during preparation.

Raw LC-MS data were preprocessed using Origin (Origin 2026 (10.3). Metabolite identities were assigned by matching MS/MS data against the HMDB and MetaboAnalyst databases. The data matrix was uploaded to the MetaboAnalyst cloud platform for analysis. All raw data are provided in Supporting Information Excel Tables S7 and S8.

### 2.12. Acquisition of Components and Disease Targets

The components identified through UPLC-MS-based untargeted metabolomics were imported into the Traditional Chinese Medicine Systems Pharmacology (TCMSP) database [49] and evaluated based on their relative abundance, oral bioavailability (OB) ≥ 20%, and drug-likeness (DL) ≥ 0.1. The processed compounds were then separately input into the PubChem database [50] to obtain canonical SMILES numbers. These SMILES strings were used to search SwissTargetPrediction [51], ChEMBL [52] and CTD (Comparative Toxicogenomics Database) [53] databases to collect targets associated with each component with a probability value of *p* > 0.1.

Disease targets were obtained by searching the keyword “gut immune modulation, Protection of colonic mucosa, ulcerative Colitis” in the GeneCards [54] database. Targets collected from each database were merged, and duplicates were removed to establish the disease target dataset. All raw data are provided in Supporting Information Excel Table S1.

### 2.13. Construction of the Protein–Protein Interaction (PPI) Network and the Component–Target–Disease Network

The targets of *S. gracilis* extract and the targets of ulcerative colitis were plotted on a Venn diagram using the Venny tool (Oliveros, J.C (2007) [55]. The resulting overlapping targets were then imported into the STRING database [56] to acquire the PPI network. Subsequently, this network was imported into Cytoscape 3.9.1 software [57] for visualization. Core targets were extracted using the CytoHubba plugin within Cytoscape. Based on the obtained component targets and their intersection with the disease targets, a component–target–disease network was constructed using Cytoscape 3.9.1 software.

### 2.14. GO Functional Annotation and KEGG Pathway Enrichment Analysis

The overlapping targets were imported into the DAVID database for Gene Ontology functional annotation and KEGG (Kyoto Encyclopedia of Genes and Genomes) pathway enrichment analysis, with the species set to “Homo sapiens” and a significance threshold of *p*-value < 0.05. Subsequently, the microbial platform was used to generate enrichment maps for GO functions and KEGG pathways, with a significance threshold of *p* < 0.05.

### 2.15. Statistical Analysis

All raw data were analyzed and visualized using GraphPad Prism 10.5.0 software. Partial data were analyzed and visualized on the Myvi Metabolism platform. For animal phenotypic data including body weight, disease activity index, organ coefficients, and colon length, and for molecular biology data including ELISA, Western blot, and immunofluorescence quantification, one-way ANOVA followed by Tukey‘s post hoc test was used for multiple-group comparisons. Two-way ANOVA with repeated measures was used for body weight change and disease activity index scores over time. For 16S rRNA and ITS sequencing data, alpha diversity indices were compared using one-way ANOVA, and beta diversity was assessed using principal coordinate analysis and permutational multivariate analysis of variance. For metabolomics data, orthogonal partial least squares discriminant analysis and univariate analysis were performed. All data are presented as mean ± standard deviation. Statistical significance was set at *p* < 0.05.

Normality and homogeneity of variance assumptions were not formally tested. The statistical methods were selected based on the experimental design and previous studies. All data are expressed as mean ± standard deviation, and the variance between groups was considered similar. Effect sizes and confidence intervals were not calculated for the outcome measures in this study. No formal study protocol was prepared or registered before the start of this experiment. The research question, experimental design, and analysis plan were determined prior to data collection based on the literature and preliminary experiments.

## 3. Results

### 3.1. Effect of PP Intervention on the Murine Ulcerative Colitis Model

We used the DSS-induced colitis model, a common model for studying colitis [58]. Figure 1A shows the experimental design. Compared with the BC group, mice in the MC group showed clear changes in body weight and DAI score, as Figure 1B,C show. Oral PP treatment clearly reduced these colitis signs, helped the mice recover, and had a dose-dependent effect.

We also checked the fecal pellets at the end of the experiment. Figure 1G shows that fecal morphology differed a lot among groups. The PP-L and PP-H groups had better fecal size and consistency than the MC group, which suggests that PP improved the intestinal condition.

After the experiment, we measured colon length. The MC group had much shorter colons than the BC group, and PP treatment largely prevented this shortening, as shown in Figure 1H,I.

We also weighed the spleen and liver. The model group had heavier spleens and livers. The PP-H group had a stronger protective effect than the PP-L group, as Figure 1E–G show.

We also performed H&E staining. Figure 1I shows the results. DSS caused severe intestinal lesions, with disrupted mucosal structure, fewer goblet cells, and clear inflammatory cell infiltration. These changes match the typical features of the colitis model. In the treatment groups, the mucosal damage and inflammatory infiltration were less severe than in the MC group, and the high-dose group showed the strongest improvement.

### 3.2. PP Ameliorates Gut Microbial Dysbiosis in Mice with Colitis

**Figure 2:**
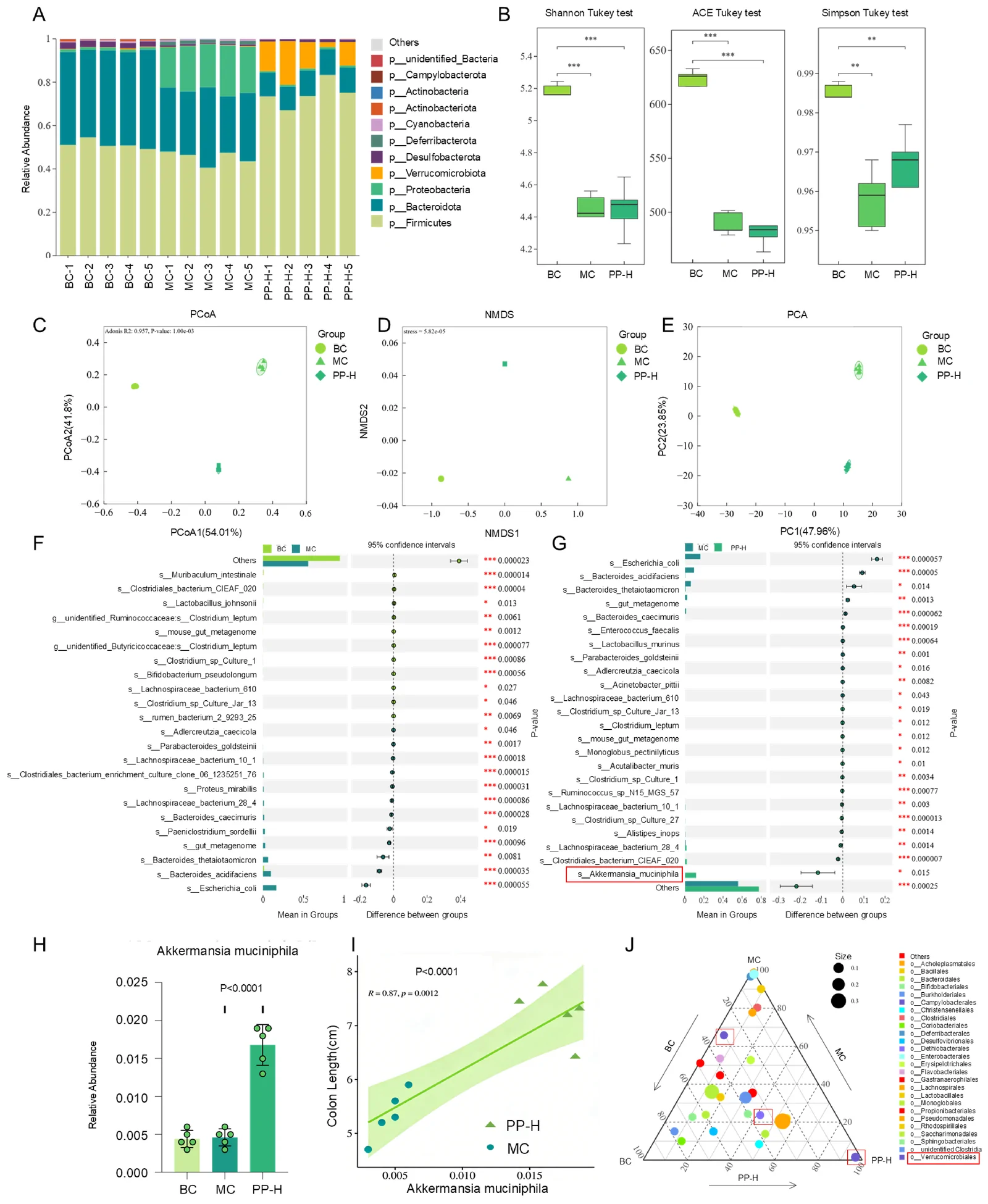
16S rRNA sequencing results of mouse intestinal contents. (**A**) Stacked bar chart of gut bacterial composition at the phylum level (*n* = 6). (**B**) Alpha diversity index analysis (*n* = 5), including Shannon, ACE, and Simpson indices. (**C**–**E**) Beta diversity analyses (PCA and NMDS). (**F**,**G**) Differential species analysis between different groups. The red box marks the position of *Akkermansia muciniphil* (**H**) Relative abundance of AKK in different groups. (**I**) Correlation analysis between AKK relative abundance and colon length in different groups. (**J**) Relative abundance of gut microbiota at the order level in each group (BC, MC and PP-H). The top 25 most abundant orders are shown, and the remaining taxa are grouped as “Others”. The red box marks the position of *Verrucomicrobiales*. Data are presented as mean ± SD (*n* = 5 per group). Data in B were analyzed by one-way ANOVA. * *p* = <0.1, ** *p* = <0.01, *** *p* = 0.001.

**Figure 3:**
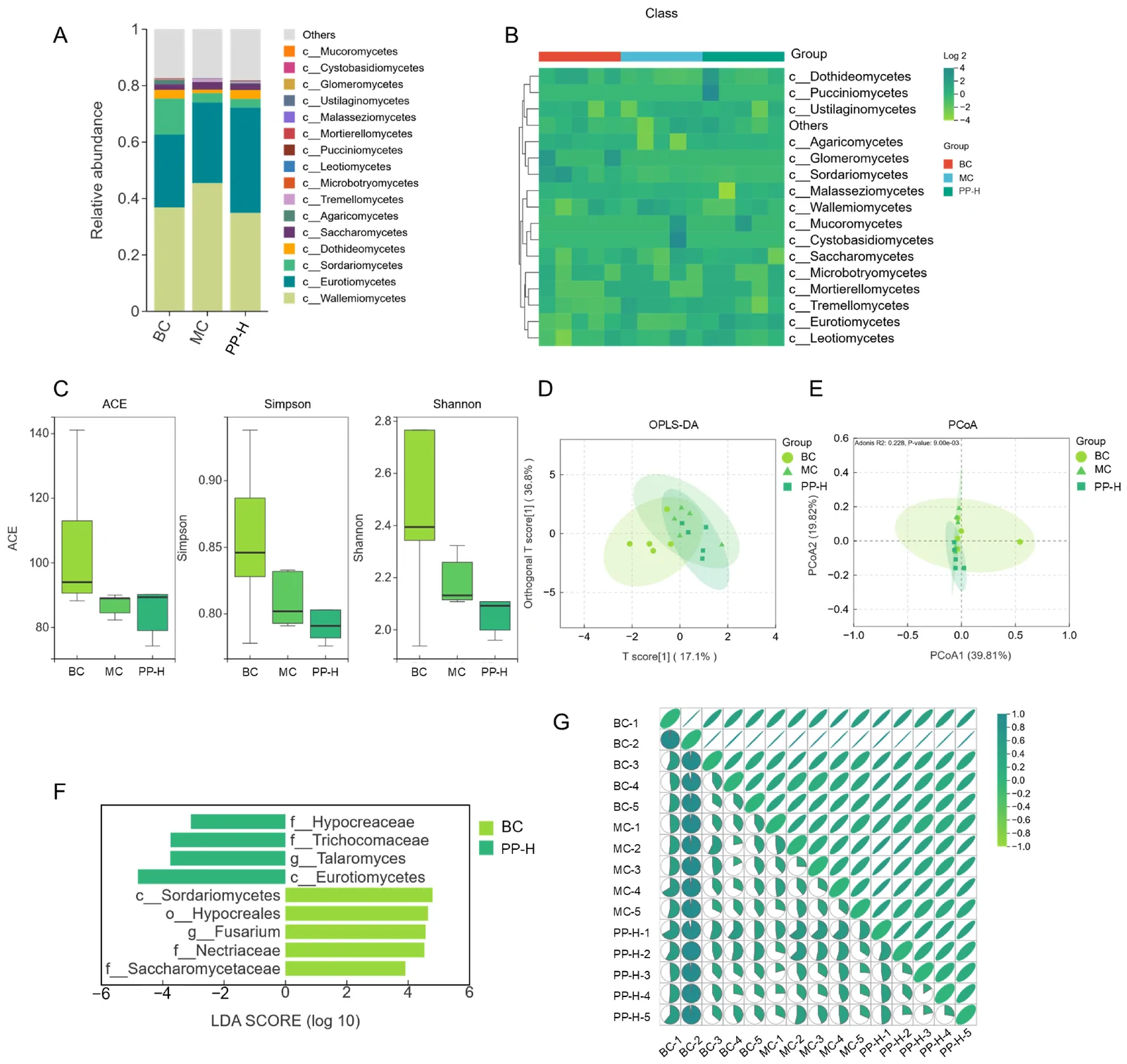
Effect of PP intervention on the intestinal fungal community structure in colitic mice. (**A**) Stacked bar chart of fungal species composition at the class level. (**B**) Heatmap of fungal species abundance at the class level. (**C**) Alpha-diversity indices (ACE, Simpson, Shannon) of the fungal community. (**D**,**E**) OPLS-DA and PCoA analyses of the fungal community. (**F**) Differential fungal species identified by LEfSe analysis. (**G**) Between-group difference analysis of beta-diversity index calculated using the Bray–Curtis distance algorithm.

Colitis does not only damage the intestine itself. The gut microbiota also play a role in making the disease hard to treat. To see how PP affects the gut microbiota in colitic mice, we collected intestinal contents at the end of the experiment and ran 16S rRNA and ITS sequencing. The sequencing results are presented in Figure 2 and Figure 3.

At the phylum level, Firmicutes and Bacteroidota dominated across all groups, together making up more than 80% of the sequences. The MC group showed a clear shift in phylum composition compared with the BC group. After PP-H treatment, the microbial composition started to move back toward normal, with more Bacteroidota and less Firmicutes, as Figure 2A shows.

When we looked at alpha diversity, the Shannon, ACE, and Simpson indices were all much lower in the MC group than in the BC group, with *p* values below 0.01 or 0.001. This means the colitis model reduced both the diversity and richness of the gut microbiota. PP-H treatment raised microbial diversity and richness, and the Shannon and ACE indices were clearly higher than those in the MC group. These results suggest that PP-H can improve the disrupted gut microbiota diversity in colitic mice.

PCoA, NMDS, and PCA based on Bray–Curtis distance all gave us a similar picture. The groups separated clearly from one another. BC samples clustered together, while MC samples sat far apart from BC, confirming that colitis disrupted the gut microbiota structure. PP-H samples moved away from MC and partly toward BC, which suggests that the PP treatment pushed the microbiota back toward a normal state. These results are shown in Figure 2C–E.

We then compared the differential taxa between groups. Compared with BC mice, the MC group had much lower abundances of several beneficial bacteria, including Lactobacillus johnsonii and *Bifidobacterium pseudolongum*, and higher levels of *pathogenic bacteria* like *Escherichia coli*. After PP-H treatment, the abundance of the species Akkermansia muciniphila, AKK for short, went up significantly, while some pathogenic bacteria went down. It is worth noting that AKK became the dominant microbial population in the PP-treated groups. These results are presented in Figure 2F–H.

Spearman correlation analysis showed a positive relationship between AKK abundance and colon length across the groups, as Figure 2I shows. The ternary plot in Figure 2J gave a straightforward view of how species abundance was distributed among the treatment groups. AKK was clearly enriched in the PP-H group, while some pathogenic bacteria were enriched in the MC group. This further supports the idea that PP treatment specifically shifted key bacterial populations and helped reshape the gut microbiota in colitic mice.

### 3.3. Effect of PP Intervention on the Intestinal Fungal Community Structure in Colitic Mice

We looked at the fungal community structure in the gut. Across all groups, *Sordariomycetes*, *Eurotiomycetes*, and several other taxa were the dominant fungal classes. Compared with the BC group, the MC group showed a clear shift in fungal composition, while the PP-H group partially restored the community structure. These changes can be seen in Figure 3A,B. When we checked alpha diversity, the ACE, Simpson, and Shannon indices were all much lower in the MC group. This tells us that colitis reduced both the diversity and richness of the gut fungi, and the PP-H treatment helped improve this dysbiosis, as shown in Figure 3C.

We then ran OPLS-DA and PCoA to compare the overall fungal community structures. The three groups separated clearly from each other. The MC group was quite different from the BC group, while the PP-H group moved partly back toward the BC group, which suggests that the PP treatment had a real effect on the fungal community. These patterns are shown in Figure 3D,E. LEfSe analysis picked out some differential fungal taxa, including *Hypocreaceae* and *Trichocomaceae*. Changes in these groups may be related to how colitis develops and responds to treatment, as we can see in Figure 3F. Correlation analysis also showed that the interaction patterns among fungal species differed between groups. This suggests that PP may improve the disrupted fungal community in colitic mice by acting on key fungal taxa and the connections between them. These results are presented in Figure 3G.

### 3.4. Effects of PP on the Intestinal Metabolomics of Colitic Mice

**Figure 4:**
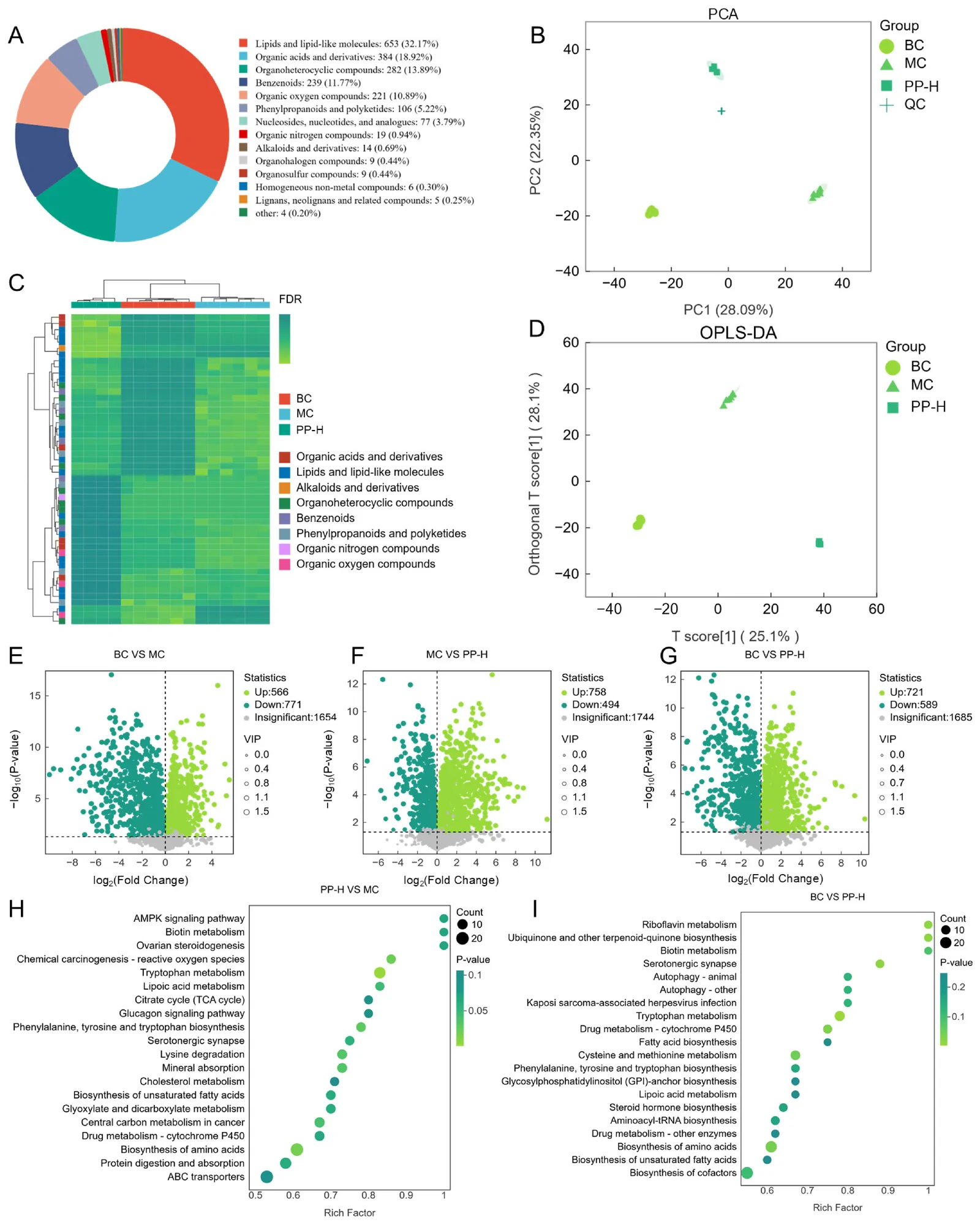
Effects of PP on the intestinal metabolomics of colitic mice. (**A**) Pie chart of metabolite classification of all identified metabolites. (**B**) Principal component analysis (PCA) score plot. (**C**) Clustering heatmap of differential metabolites. (**D**) Orthogonal partial least squares discriminant analysis (OPLS-DA) score plot. The notations [1] indicate the first predictive component and the first orthogonal component of the OPLS-DA model, respectively. (**E**–**G**) Volcano plots of differential metabolites between groups (BC vs. MC, MC vs. PP-H, BC vs. PP-H). The horizontal dotted line indicates the significance threshold of *P* = 0.05, and the vertical dotted lines indicate the log_2_(Fold Change) threshold for screening differential metabolites. (**H**,**I**) Bubble plots of KEGG pathway enrichment analysis for differential metabolites. QC, quality control samples.

DSS colitis does not just change the microbes in the gut contents; it also causes inflammation in the intestine itself. So, at the end of the experiment, we collected intestinal tissues to see how the metabolism had been affected, and these results are presented in Figure 4.

Looking at the metabolomics data, we found that the metabolites in the gut contents were mostly lipids and lipid-like molecules, organic acids and their derivatives, and a few other classes, as Figure 4A shows. The PCA and OPLS-DA score plots in Figure 4B,D revealed a clear separation among the BC, MC, and PP-H groups. The MC group showed a serious disruption of the intestinal metabolic profile, while the PP-H treatment helped bring it back toward normal. When we compared the differential metabolites, Figure 4E,G show that many metabolites changed significantly in the MC group compared with the BC group, and the PP-H treatment reversed several key ones back toward normal levels. We then ran a KEGG pathway enrichment analysis, and the results in Figure 4H,I point to lipid metabolism, amino acid metabolism, and bile acid metabolism as the main affected pathways. These findings suggest that PP may protect mice from colitis by reducing the metabolic disturbances in the gut and helping restore energy and substance metabolism.

### 3.5. Network Pharmacology Analysis Indicates That PP May Inhibit the AGE/RAGE Pathway

**Figure 5:**
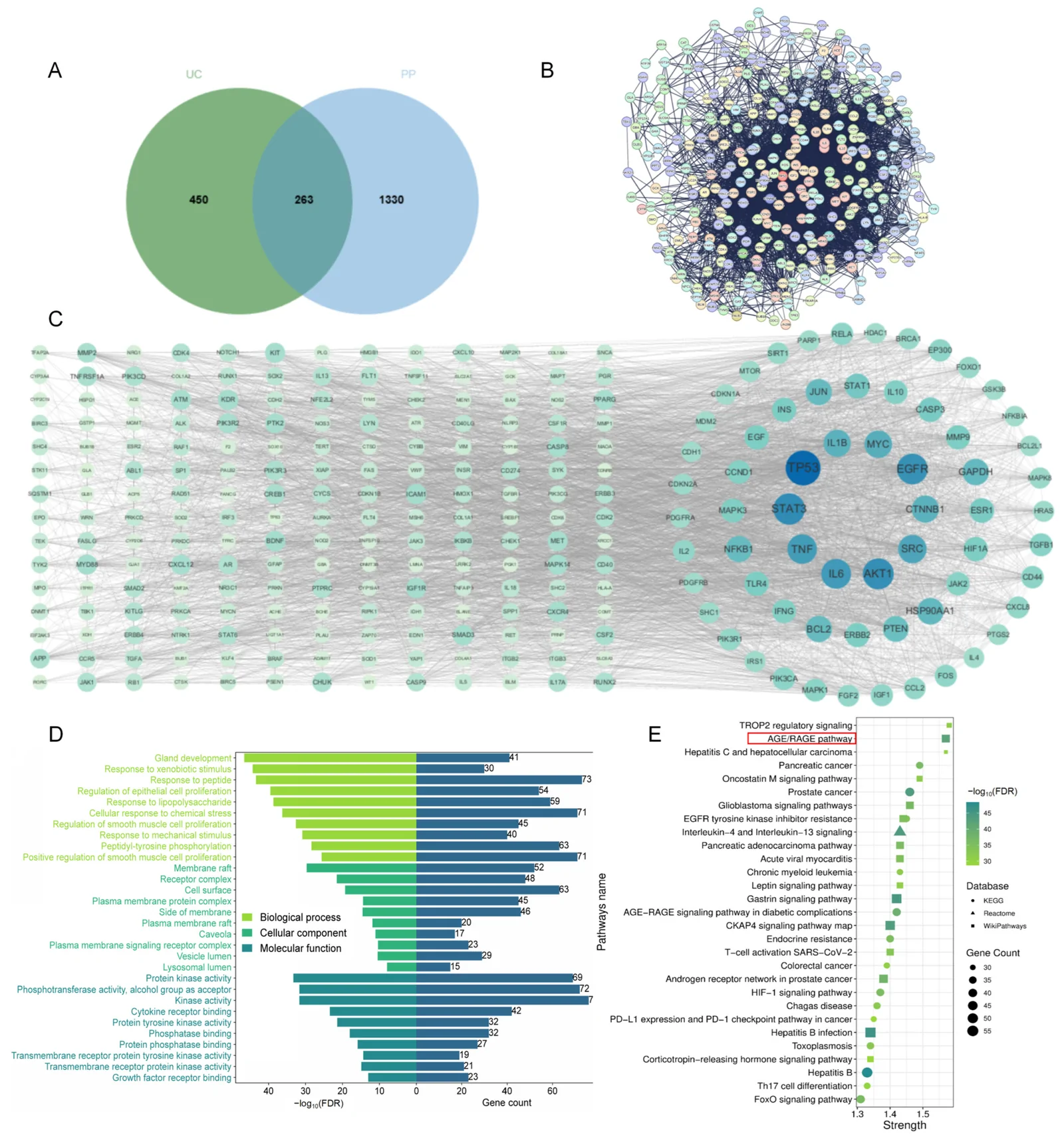
Network pharmacology analysis indicates that PP may inhibit the AGE/RAGE pathway. (**A**) Venn diagram of shared targets between ulcerative colitis and PP active components. (**B**) Protein–protein interaction (PPI) network constructed for the intersecting targets. (**C**) Visualization of core hub targets identified by Cytoscape analysis. (**D**) Bar chart of Gene Ontology (GO) functional enrichment analysis. (**E**) Bubble chart of pathway enrichment analysis based on multi-database retrieval. The red box highlights the AGE/RAGE pathway, one of the major enriched signaling pathways in this enrichment analysis plot.

We wanted to figure out how PP might work against ulcerative colitis, so we first looked at the overlapping targets between UC and the active components of PP. Figure 5A shows the Venn diagram, and we got 263 potential common targets. From these, we built a PPI network, shown in Figure 5B,C. Topological analysis then picked out several core targets, including TP53, STAT3, TNF, IL-6, AKT1, and EGFR. These are mostly well-known molecules involved in inflammation and cell proliferation, and they may be the key nodes through which PP acts. Next, we ran GO enrichment analysis on these overlapping targets, and Figure 5D shows the results. The targets were mainly linked to biological processes like the regulation of inflammatory responses and cellular responses to chemical stimuli. At the molecular level, they were connected to protein kinase activity and receptor binding. Most of them were also located in the cell membrane, which fits with what we know about intestinal inflammation. When we looked at pathway enrichment across multiple databases, the AGE-RAGE signaling pathway stood out, as shown in Figure 5E. These findings suggest that PP may reduce inflammation and help repair intestinal damage by acting on these core targets and pathways. This gives us a clear direction for the next round of mechanistic experiments.

### 3.6. Effect of PP on the AGE/RAGE Pathway and Inflammatory Factor Expression in Colonic Tissues of Colitic Mice

**Figure 6:**
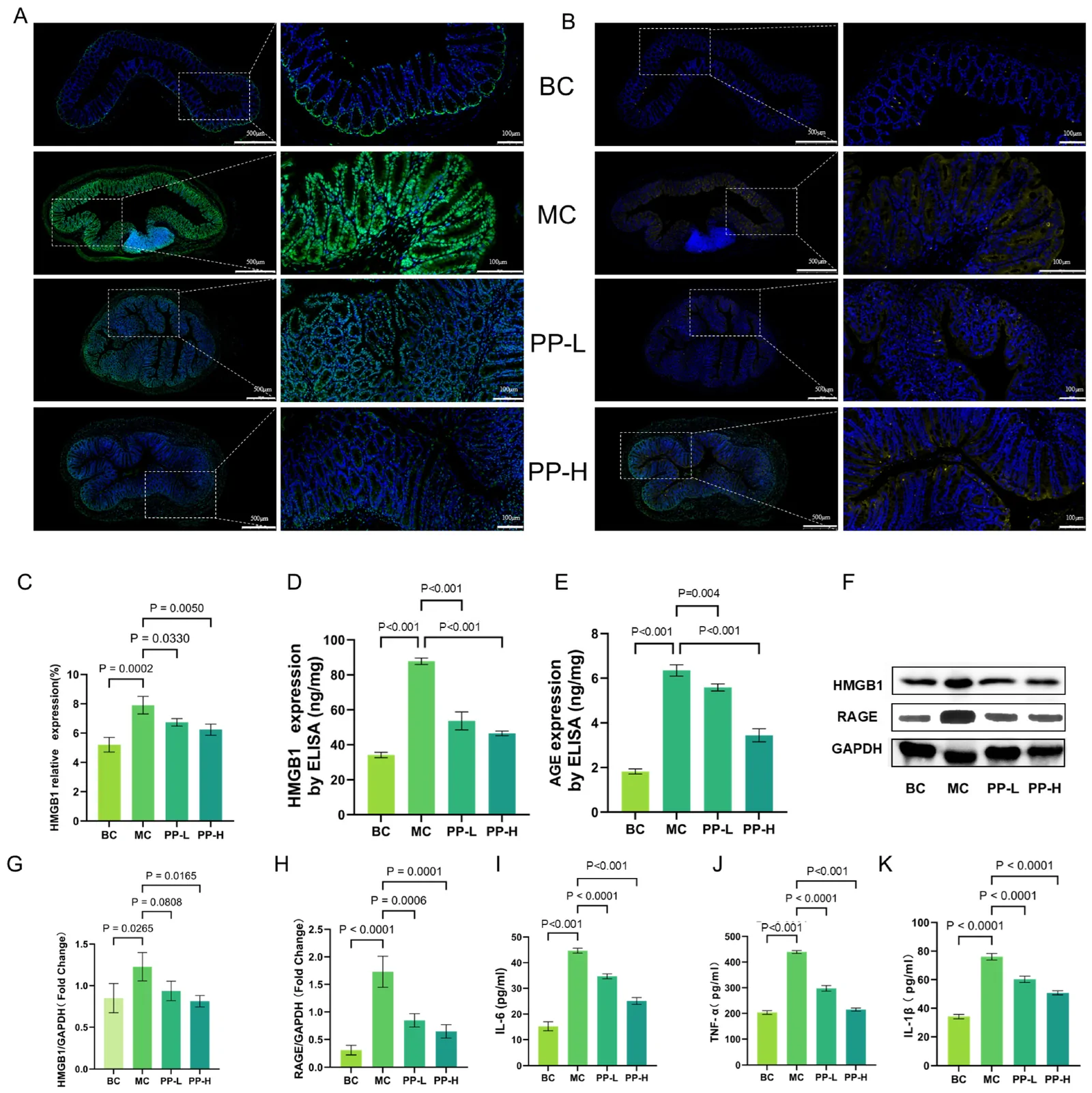
Effect of PP on the AGE/RAGE pathway and inflammatory factor expression in colonic tissues of colitic mice. (**A**) Immunofluorescence staining of HMGB1 in colon tissue. The dotted lines outline representative regions, and the corresponding magnified views are presented on the right of each full-field image. (**B**) Immunofluorescence staining of RAGE in colon tissue. The dotted lines outline representative regions, and the corresponding magnified views are presented on the right of each full-field image. (**C**–**E**) Relative expression levels of HMGB1, AGE, and RAGE in colon tissue. (**F**–**H**) Western blot detection of HMGB1 and RAGE protein expression and densitometric analysis. (**I**–**K**) Serum levels of the inflammatory cytokines IL-6, TNF-α, and IL-1β measured by ELISA. Data is presented as mean ± SD.

Figure 6A shows the immunofluorescence staining results. In the MC group, the fluorescence signals of HMGB1 and RAGE in colonic tissues were much stronger than those in the BC group, indicating clear activation of the AGE/RAGE pathway. PP-H and PP-L treatment significantly reduced these signals, which suggests that PP can suppress this pathway. Quantitative analysis showed that HMGB1, RAGE, and AGE levels in colonic tissues were significantly higher in the MC group than in the BC group, and serum IL-6, TNF-α, and IL-1β were also clearly elevated. After PP treatment, the levels of these pathway molecules and pro-inflammatory cytokines dropped in a dose-dependent manner, with the strongest effect in the PP-H group. Together, these results indicate that PP protects mice against colitis by blocking the AGE/RAGE pathway and reducing the release of pro-inflammatory cytokines.

### 3.7. Effect of PP on Colonic Histopathology and Related Indicators in DSS-Induced Colitic Mice

**Figure 7:**
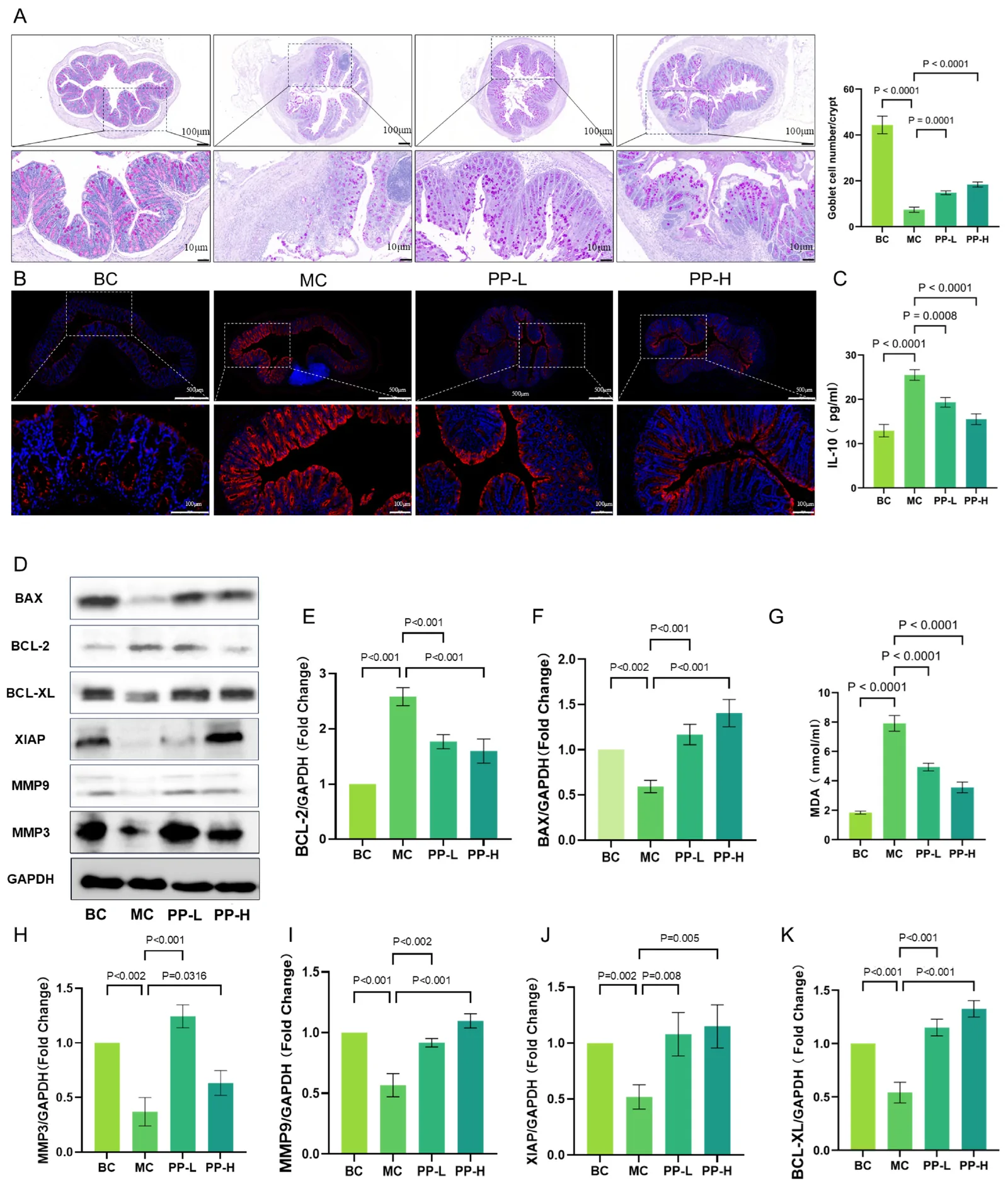
Effect of PP on colonic histopathology and related indicators in DSS-induced colitic mice. (**A**) H&E staining and goblet cell count in mouse colonic tissue. (**B**) Immunofluorescence staining results of mouse colonic tissue. (**C**) IL-10 levels in colonic tissue. (**D**–**K**) Protein expression levels and densitometric analysis of apoptosis-related proteins (BAX, BCL-2, BCL-XL, XIAP), matrix metalloproteinases (MMP3, MMP9), and the oxidative stress marker MDA. Data are presented as mean ± SD.

PP intervention significantly reversed the elevated IL-10 level observed in the MC group. Figure 7A shows the histopathological changes and related markers in the mouse colon tissues. Compared with the BC group, the MC group had severe disruption of the mucosal structure and a clear loss of goblet cells. After PP treatment, both the PP-L and PP-H groups showed less mucosal damage, and goblet cells recovered in a dose-dependent manner, with the strongest recovery in the PP-H group. We next examined the immunofluorescence staining results shown in Figure 7B. The MC group had much stronger fluorescence signals for inflammation-related markers in the colon, while PP treatment reduced these signals, suggesting less inflammatory infiltration. As for IL-10, the ELISA results shown in Figure 7C indicated that its level was higher in the MC group, and PP brought this level back down. Figure 7D,K shows the Western blot and densitometry results. Compared with the BC group, the MC group had higher expression of the pro-apoptotic protein BAX, the pro-inflammatory proteins MMP3 and MMP9, and the oxidative stress marker MDA, while the anti-apoptotic proteins BCL-2, BCL-XL, and XIAP were lower. PP reversed these changes by lowering BAX, MMP3, MMP9, and MDA, and raising BCL-2, BCL-XL, and XIAP, with stronger effects in the high-dose group. Taken together, PP reduces colonic tissue damage in colitic mice by suppressing inflammation, oxidative stress, and the changes in apoptosis-related proteins.

## 4. Discussion

The DSS-induced mouse colitis model is widely used in colitis research. It causes weight loss, diarrhea, bloody stools, and obvious colon shortening. These symptoms are hard to reverse. In this study, we found that oral PP markedly reduced the severity of DSS-induced colitis. Notably, PP not only helped restore the gut microbiota in the intestinal contents but also acted directly on the intestine itself.

**Figure 8:**
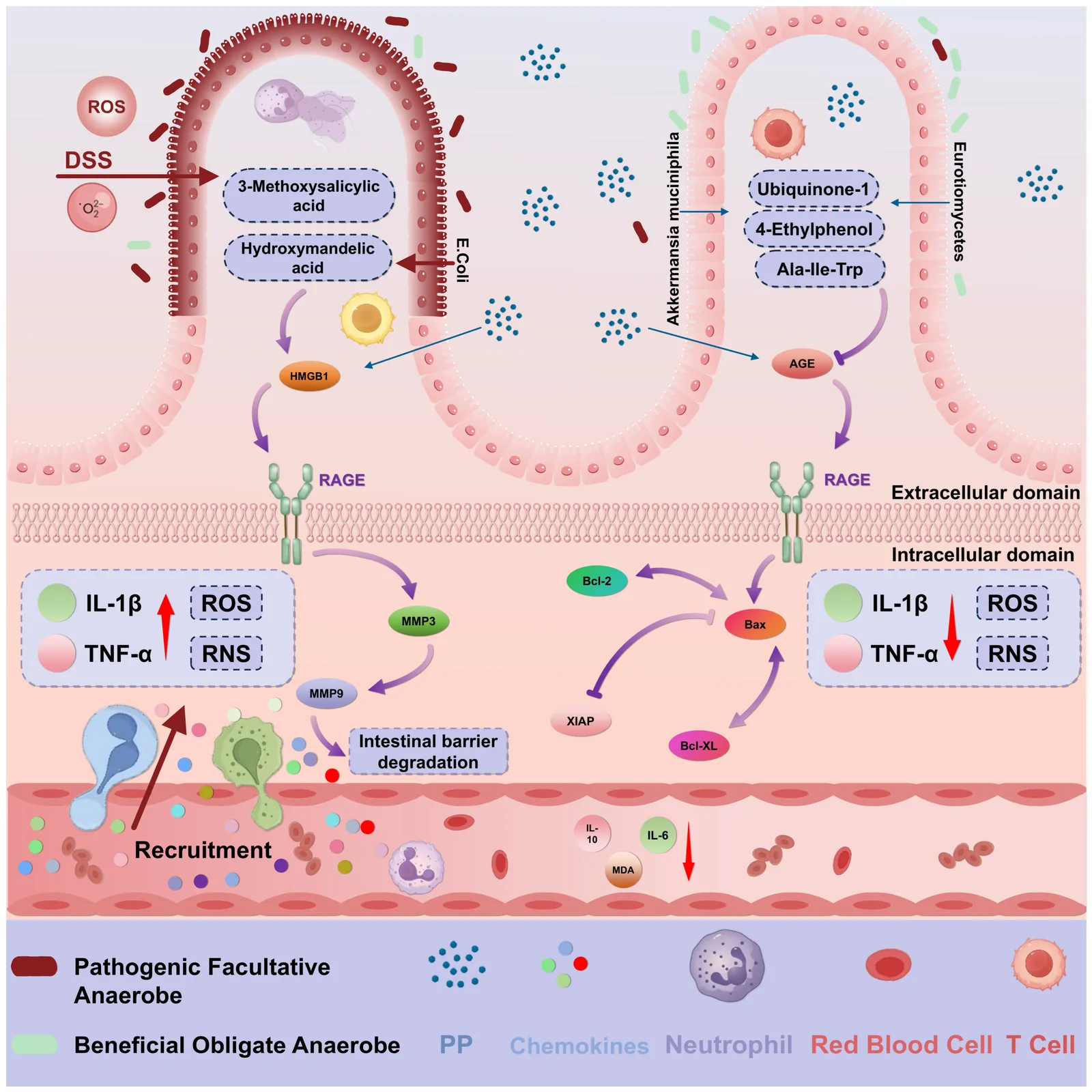
Schematic diagram of the mechanism. Partial drawing materials provided by Figdraw (Figdraw 2.0). In this schematic diagram, blue arrows indicate the movement direction of substances. Purple arrows represent promoting regulatory effects, while purple flat-headed arrows indicate inhibitory regulatory effects. Red arrows denote the upregulation or downregulation of inflammatory factors, oxidative stress indicators and other related molecules.

This study shows that PP can regulate the AGE/RAGE signaling pathway. It does this by modulating the expression of MMP9, MMP3, and the pro-apoptotic factor BAX. This protects the intestine and reduces oxidative stress and apoptosis in colonic tissue. At the same time, PP restores goblet cell numbers and protects the intestinal mucosal barrier. This significantly alleviates DSS-induced mouse colitis. PP also has clear anti-inflammatory, antioxidant, and microecological modulatory effects. It is a promising natural-derived, multi-target candidate drug for relieving ulcerative colitis. Based on the above experimental results, we propose the underlying regulatory mechanism, which is visually summarized in Figure 8.

PP significantly restored AKK abundance and improved tryptophan and biotin metabolism [59,60,61], reflecting a modulation of microbial metabolic output beyond mere compositional changes. Unlike previous studies focusing on NF-κB or MAPK, we identified the AGE-RAGE axis as a key anti-inflammatory target of PP via network pharmacology and experimental validation, with PP downregulating HMGB1 and blocking RAGE activation. This multi-target mode, together with goblet cell protection and apoptosis regulation, distinguishes PP from conventional immunosuppressive strategies.

In this study, through integrative analysis of multi-omics data and experimental validation, we systematically elucidated the underlying biological mechanisms by which PP alleviates UC. At the macroscopic level, based on phenotypic data from mice, PP significantly inhibited DSS-induced body weight loss and the increase in DAI score, and alleviated colon shortening.

The protective effects of PP were accompanied by changes in the gut microbiota. Gut dysbiosis is an important driver of UC, and AKK is known to support mucus layer integrity and promote an anti-inflammatory microenvironment [62]. The enrichment of AKK by PP therefore contributes to mucosal protection. The positive correlation between AKK abundance and colon length further suggests that restoration of this keystone species is closely associated with improved intestinal function. In addition, PP remodeled the fungal community, which may have reduced the overgrowth of pro-inflammatory fungi and rebalanced bacterial–fungal interactions, thereby alleviating a previously underappreciated source of intestinal inflammation.

These microbial changes were accompanied by metabolic alterations, providing a mechanistic link between the gut microbiota and host immunity. PP corrected disturbances in tryptophan, biotin, and AMPK-related pathways. Tryptophan metabolites act as endogenous ligands of the aryl hydrocarbon receptor (AhR), playing a critical role in maintaining epithelial barrier integrity and inducing immune tolerance [63]. Therefore, restoration of tryptophan metabolism may represent an important route through which PP enhances mucosal defense. The enrichment of AMPK-related pathways further suggests that PP improved cellular energy homeostasis and reduced oxidative stress, creating a more favorable environment for epithelial repair [64].

The AGE-RAGE axis serves as an important bridge between oxidative stress and persistent inflammation [65]. PP downregulated HMGB1 and RAGE, which blocks the feed-forward loop in which extracellular HMGB1 activates RAGE and amplifies the release of TNF-α, IL-6, and IL-1β [66]. The reduced levels of these pro-inflammatory cytokines in colonic tissues indicate that blockade of this axis is one of the key mechanisms underlying the anti-inflammatory effects of PP. The restoration of goblet cells and the modulation of apoptosis-related proteins and MMPs explain the structural protection of the mucosa. By increasing goblet cells, PP supported mucus production and barrier integrity; by regulating Bcl-2 family proteins, it reduced excessive apoptosis of epithelial cells under inflammatory stress; and by lowering MMP3 and MMP9 levels, it limited extracellular matrix degradation. Collectively, these effects on the gut microbiota, metabolism, inflammatory signaling, and the mucosal barrier account for the protective effects observed in DSS-induced colitis.

Our study has several limitations. We only used the DSS-induced acute colitis model, which does not fully reflect the chronic and relapsing features of human UC. In addition, the multi-omics findings are correlative rather than causal; FMT or mono-colonization studies are needed to confirm whether AKK enrichment directly contributes to the therapeutic effects. Notably, since its discovery in 2004, AKK has been widely studied in colitis. Oral AKK administration [67,68], AKK supplementation under psychological stress [61], and FMT from healthy donors [69] have all been reported to alleviate colitis. Similarly, many interventions, including puerarin [70], gastrodin [71], metformin [72], peimine [73], and several plant extracts [74,75,76,77], have been shown to increase AKK abundance. These findings suggest that AKK may be a common microbial responder in colitis rather than a specific mediator of PP.

One limitation is that we did not look at PP’s pharmacokinetics in vivo, so we do not know whether PP acts directly on host receptors or works through metabolites produced by gut microbes. Future work could use drug metabolism tracing and gene knockout models to clarify this. Even so, these limitations do not undermine our main findings, but rather suggest directions for further study. Our results still support PP as a potential therapeutic option for UC through its effects on the gut microecology.

This study showed how PP alleviates colitis by repairing the mucosal barrier, reshaping gut bacteria and fungi, and inhibiting the AGE/RAGE pathway. These results add to what we know about UC. However, some limitations and practical challenges remain. The DSS model can mimic inflammatory injury, but it does not fully reflect the chronic and relapsing nature of human UC. Also, the causal link between microbial changes and therapeutic effects has not been directly confirmed, for example, by fecal microbiota transplantation. On the theoretical side, our study challenges the previous focus on bacteria alone and provides evidence supporting a role for the fungal community in protecting the intestinal barrier. This study suggests that the anti-inflammatory and metabolic regulatory functions of PP provide potential possibilities for its development as a microecological preparation or a food for special medical purposes. However, this application prospect still needs to be validated by rigorous randomized double-blind clinical trials targeting the AGE/RAGE pathway. Current results are derived from preclinical studies, and commercial translation requires additional data support.

## 5. Conclusions

This study demonstrated that PP significantly alleviated DSS-induced acute colitis in mice, as evidenced by reduced DAI scores, attenuated body weight loss, restored colon length, and normalized IL-10 levels. PP reversed DSS-induced bacterial–fungal dysbiosis, markedly increased the relative abundance of AKK, and restored metabolic homeostasis of lipids, organic acids, and aromatic compounds. PP inhibited the AGE/RAGE signaling pathway, reduced MDA accumulation, and modulated apoptosis-related proteins and matrix metalloproteinases, thereby protecting the intestinal mucosal barrier and restoring goblet cells. These findings provide scientific evidence for PP as a potential gut microecological modulator in IBD.

## Data Availability

The original contributions presented in the study are included in the Supplementary Material. Further inquiries can be directed to the corresponding authors.

## Supplementary Materials

The following supporting information can be downloaded at: https://www.mdpi.com/article/10.3390/antiox15091130/s1, SI Excel Table S1 Raw data on diseases and components(PP) targets; SI Table S2 DAI scoring criteria; SI Table S3 The Gradient Elution Process of PP Samples; SI Table S4 The Mass Spectrum Conditions of All PP Samples; SI Table S5 The Gradient Elution Process of tissue samples; SI Table S6 The Mass Spectrum Conditions of All tissue Samples; SI Excel Table S7 Raw LC-MS data of all tissues; SI Excel Table S8 Raw LC-MS data of PP tissues; SI Table S9 Antibody Usage Information; SI Figure S1. Differential metabolite Bar plots under three conditions; SI Figure S2. Representative LC-MS total ion chromatograms (TICs) of PP samples; SI Figure S3. Overlaid total ion chromatograms (TICs) of quality control (QC) samples in negative and positive ionization modes.

## Abbreviations

AGE	Advanced glycation end products
RAGE	Receptor for advanced glycation end products
HMGB1	High mobility group box 1
IL-1β	Interleukin-1 beta
TNF-α	Tumor necrosis factor alpha
IL-6	Interleukin-6
IL-10	Interleukin-10
MDA	Malondialdehyde
BAX	Bcl-2-associated X protein
Bcl-2	B-cell lymphoma 2
Bcl-xL	B-cell lymphoma-extra large
XIAP	X-linked inhibitor of apoptosis protein
MMP3	Matrix metalloproteinase 3
MMP9	Matrix metalloproteinase 9
AKK	*Akkermansia muciniphila*
H&E	Hematoxylin and eosin
PAS	Periodic acid-Schiff
16S rRNA	16S ribosomal RNA
ITS	Internal transcribed spacer
*S. gracilis*	*Sphallerocarpus gracilis* (Besser ex Trevir.) Koso-Pol
PP	*Sphallerocarpus gracilis* root polyphenols extract
E-cadherin	Epithelial cadherin
