# Supplementary material for: Sphallerocarpus gracilis Polyphenols Alleviate DSS-Induced Colitis by Remodeling Gut Microbiota and Inhibiting the AGE-RAGE/HMGB1 Pathway
Source: Antioxidants (Basel). 2026 Sep 7;15(9):1130. doi: 10.3390/antiox15091130 (PMC13603434; doi:10.3390/antiox15091130)
Supplement: Supplementary file 1 [file antioxidants-15-01130-s001.zip › antioxidants-4462072-supplementary.pdf]

Supporting Information

Table of Contents

Supporting Information Excel Table S1 Raw data on diseases and components(PP) targets 1

Supporting Information Table S2 DAI scoring criteria. 1

Supporting Information Table S3 The Gradient Elution Process of PP Samples 2

Supporting Information Table S4 The Mass Spectrum Conditions of All PP Samples..... 3

Supporting Information Table S5 The Gradient Elution Process of tissue samples. 5

Supporting Information Table S6 The Mass Spectrum Conditions of All tissue Samples. 5

Supporting Information Excel Table S7 Raw LC-MS data of all tissues. 5

Supporting Information Excel Table S8 Raw LC-MS data of PP tissues. 6

Supporting Information Table S9 Antibody Usage Information 6

Supporting Information Figure S1. Differential metabolite Bar plots under three conditions. 7

Supporting Information Figure S2.Representative LC-MS total ion chromatograms (TICs) of PP samples. 8

Supporting Information Figure S3 Overlaid total ion chromatograms (TICs) of quality control (QC) samples in negative and positive ionization modes. 9

Supporting Information Excel Table S1 Raw data on diseases and components(PP) targets

NO.	Disease Trage	Components （PP）-Target
1	TP53	GYS2
2	ATM	IRS1
3	PALB2	PCK1
4	CHEK2	PTPN1
5	BRCA2	PYGL
6	BRIP1	NFE2L2
7	APC	AKT
8	BRCA1	GSK3B
9	MLH1	HMOX1
10	PTEN	INSR

This table is an embedded Excel object within the Word file. Double-click it to see full contents when the document is opened in Word.

Supporting Information Table S2 DAI scoring criteria.

Score	Weight loss	Stool consistency	fecal blood
0	< 2%	Normal	Negative
1	2%–5%	Loose stool	Postive
2	5%–10%		
3	10%–15%		
4	≥15%	Watery stool	Gross hematochezia

For the obtained PP sample, 50 mg of the powder was weighed using an electronic balance and transferred to a tube, after which 1,200 μL of pre-cooled (−20 °C) 70% methanol aqueous solution containing internal standards was added. The internal standard solution was prepared by first dissolving 1 mg of standard in 1 mL of 70% methanol to obtain a 1,000 μg/mL stock solution, which was then further diluted with 70% methanol to yield a 250 μg/mL internal standard working solution. The mixture was vortexed for 30 s every 30 min, for a total of 6 vortexing cycles. After centrifugation at 12,000 rpm for 3 min, the supernatant was collected and filtered through a microporous membrane (0.22 μm pore size), then stored in an injection vial for UPLC-MS/MS analysis. The LC-MS conditions were as follows: column, Waters ACQUITY UPLC HSS T3 (1.8 μm, 2.1 mm × 100 mm); mobile phase A, ultrapure water with 0.1% formic acid; mobile phase B, acetonitrile with 0.1% formic acid; column temperature, 40 °C; flow rate, 0.40 mL/min; injection volume, 4 μL. The elution gradient is shown in Table 1. The UPLC system was interfaced with a TripleTOF 6600+ mass spectrometer. The elution gradient settings and mass spectrometry parameters are detailed in Supplementary Information Tables 3 and 4.

Supporting Information Table S3 The Gradient Elution Process of PP Samples

Time (min)	Phase A (%,v/v)	Phase B (%,v/v)
0	95	5
5	35	65
6	1	99
7.5	1	99
7.6	95	5
10	95	5

Supporting Information Table S4 The Mass Spectrum Conditions of All PP Samples

Conditions	ESI+	ESI-
Duration (min)	10	10
IonSpray Voltage (V)	5000	-4000
Temperature (°C)	550	550
Ion Source Gas1 (psi)	50	50
Ion Source Gas2 (psi)	60	60
Curtain Gas (psi)	35	35
Declustering Potential (V)	60	-60
MS1 Collision Energy (V)	10	-10
MS2 Collision Energy (V)	30	-30
Collision Energy Spread (V)	15	15
MS1 TOF Masses (Da)	50~1250	50~1250
MS2 TOF Masses (Da)	25~1250	25~1250

The intestinal tissue samples were collected from the mice, immediately snap-frozen in liquid nitrogen, and then transferred to a −80 °C freezer for storage. The frozen intestinal tissues were removed from the −80 °C freezer and thawed on ice until they were amenable to cutting; all subsequent operations were performed on ice. The tissue was minced and mixed thoroughly, and multiple aliquots were taken to weigh 20 mg of tissue into correspondingly labeled centrifuge tubes. One steel bead was added to each tube, and the tissues were homogenized using a ball mill at 30 Hz for 20 s, followed by centrifugation at 3,000 rpm for 30 s at 4 °C to pellet the samples. After centrifugation, 400 μL of 70% methanol aqueous solution containing internal standards was added, and the mixture was vortexed at 1,500 rpm for 5 min and then placed on ice for 15 min. The samples were then centrifuged at 12,000 rpm for 10 min at 4 °C, and 300 μL of the supernatant was transferred to a new centrifuge tube and left to stand at −20 °C for 30 min. Subsequently, the samples were centrifuged again at 12,000 rpm for 3 min at 4 °C, and 200 μL of the supernatant was transferred to an insert in an injection vial. The analysis was performed using a Thermo Scientific Vanquish system (Thermo Scientific, Massachusetts, USA) coupled to a Q Exactive HF-X mass spectrometer (Thermo Scientific, Massachusetts, USA), with a Waters ACQUITY Premier HSS T3 column (1.8 μm, 2.1 mm × 100 mm). Mobile phase A was 0.1% formic acid in water, and mobile phase B was 0.1% formic acid in acetonitrile. The chromatographic conditions were set as follows: column temperature 40 °C, flow rate 0.4 mL/min, and injection volume 3 μL. The LC gradient program and Q Exactive HF-X mass spectrometry conditions are shown in Supporting Information Table 5 and Table 6.

Raw data acquired by liquid chromatography-mass spectrometry (LC-MS) were preprocessed using OriginQI software (Waters, Milford, USA). Metabolite identities were assigned by matching MS and MS/MS spectra against public metabolomics databases, including HMDB and the proprietary MetaboAnalyst database. The data matrix was uploaded to the MetaboAnalyst cloud platform (www.metaboanalyst.com) for analysis. All raw data are provided in Supplementary Information Table 7.

Supporting Information Table S5 The Gradient Elution Process of tissue samples.

Time (min)	A (%)	B (%)
0	95	5
2	80	20
5	40	60
6	1	99
7.5	1	99
7.6	95	5
10	95	5

Supporting Information Table S6 The Mass Spectrum Conditions of All tissue Samples.

Conditions	ESI+	ESI-
Spray Voltage	3800	3400
Sheath gas (Arb)	60	60
Aux gas (Arb)	20	20
Ion transfer tube temperature	320	320
Vaporizer temperature	350	650
Scan Range	70-1000	70-1000
Resolution	60000	60000
AGC target	3.00E+06	3.00E+06
RF lens (%)	50	50
Scan Range	70-1000	70-1000
Resolution	15000	15000
ACG target	1.00E+05	1.00E+05
Collision energy (CE)	304,050	304,050
Signal Intensity Threshold	1.00E+06	1.00E+06
Top N vs Top speed	10	10
Exclusion duration	3	3

Supporting Information Excel Table S7 Raw LC-MS data of all tissues.

	Mode	Compounds	BC_1	BC_2	BC_3	BC_4	BC_5	BC_6	MC_1	MC_2	MC_3	MC_4	MC_5	MC_6	PP_H_1	PP_H_2	PP_H_3	PP_H_4	QC01	QC02
1	T3_positiv	Niacinamid	6.63E+09	6.8E+09	6.72E+09	6.51E+09	6.71E+09	6.51E+09	7.6E+09	7.76E+09	7.39E+09	7.46E+09	7.5E+09	7.4E+09	5.47E+09	5.45E+09	5.67E+09	5.57E+09	5.43E+09	5.42E+09
2	T3_positiv	o-1-phenyl	3.49E+09	3.3E+09	3.47E+09	3.55E+09	3.48E+09	3.26E+09	5.92E+09	6.19E+09	6.1E+09	6.31E+09	6.39E+09	6.18E+09	4.59E+09	4.27E+09	4.49E+09	4.21E+09	8.28E+09	8.22E+09
3	T3_positiv	sphorylox	26483366	31541817	29933460	28402476	29659402	28149560	72435492	71277961	78382189	68206015	80248860	69068444	31459226	26469931	34876603	29437593	1.8E+08	1.76E+08
4	T3_positiv	Methionine	3.53E+08	3.53E+08	3.37E+08	3.36E+08	3.38E+08	3.2E+08	4.86E+08	4.77E+08	5.09E+08	5.17E+08	5.11E+08	5.3E+08	4.33E+08	4.18E+08	6.41E+08	4.66E+08	6.15E+08	6.16E+08
5	T3_positiv	droxytrypt	70601643	71606123	69974864	71457687	74571159	66725277	22624322	22081521	24583424	22801535	20197661	21975059	32698435	36769782	35072302	35418731	37411589	37263313
6	T3_positiv	tyl-L-carni	5.26E+08	5.06E+08	5.11E+08	4.93E+08	4.79E+08	4.88E+08	6.7E+08	7.16E+08	6.86E+08	7.41E+08	7.08E+08	6.8E+08	1.1E+09	1.13E+09	1.11E+09	1.15E+09	6.65E+08	6.65E+08
7	T3_positiv	Proline	1.45E+09	1.32E+09	1.34E+09	1.26E+09	1.21E+09	1.16E+09	1.97E+09	1.88E+09	1.95E+09	2.01E+09	1.98E+09	1.87E+09	1.53E+09	1.49E+09	1.46E+09	1.4E+09	1.99E+09	1.97E+09
8	T3_positiv	Lysine	3139787	3367716	3222532	3218312	2619579	3139054	6267449	6522105	6746005	6468467	6394231	5749433	4193840	4675899	4048294	4816304	11750961	11656688
9	T3_positiv	Carnitine	6.21E+08	6.97E+08	7.04E+08	6.91E+08	6.49E+08	7.02E+08	7.6E+08	7.57E+08	8.03E+08	7.97E+08	9.44E+08	8.14E+08	8.59E+08	7.6E+08	8E+08	7.88E+08	7.25E+08	7.25E+08

This table is an embedded Excel object within the Word file. Double-click it to see full contents when the document is opened in Word.

Supporting Information Excel Table S8 Raw LC-MS data of PP tissues.

	Mode	Compounds	drug-like pı	oral availab	Class I	CAS	PP-1	PP-2	PP-3	QC01	QC02	QC03
1	T3_negative	Isoginkgetir	0.58	21.56	Flavonoids	548-19-6	2123510	7162187	2442763	171339.1	185447.6	187932.1
2	T3_positive	lpha-Asaron	0.06	35.61	henolic acio	2883-98-9	1122220	1094031	1183124	411740	403650.9	411100.7
3	T3_negative	ospermic ac	0.41	3.01	henolic acio	90-2 | 1159	15715.68	531972.4	384318.1	1253822	1229792	1251241
4	T3_negative	ydroxycoun	0.05	15.8	ns and Cour	6093-68-1	509820.1	520731.8	514766.2	151069.6	149142.7	149873.9
5	T3_positive	Usnic acid	0.39	5.36	henolic acio	125-46-2	498208.9	499513.4	523475.8	216404.9	216086	220424.3
6	T3_negative	roxycinnam	0.04	49.54	henolic acid	14755-02-3	379151.4	376989.5	297688.4	150548.2	148917.5	149160.5
7	T3_positive	Naringin	0.78	6.92	Flavonoids	10236-47-2	237991.7	256470.6	233931.7	93487.69	91213.12	92729.58
8	T3_negative	bavachalco	0.27	1.04	Flavonoids	50-3 | 5467	240784.3	239573.2	237435.1	84721.47	82810.54	84880.3
9	T3_negative	Luteolin	0.25	36.16	Flavonoids	491-70-3	211755.9	207396.3	205236	125121.7	125035.9	124992.4
10	T3_negative	Didymin	0.24	38.55	Flavonoids	14259-47-3	213917.3	197046	203531.9	71466.13	70179.37	71553.17

This table is an embedded Excel object within the Word file. Double-click it to see full contents when the document is opened in Word.

Supporting Information Table S9 Antibody Usage Information

Antibody Name	Dilution Ratio	Suppliers	Catalog Numbers
HMGB1	1:1000	Servicebio, Wuhan, China	V2.0-20251020
E-cadherin	1:1000	Servicebio, Wuhan, China	V2.0-20250849
RAGE	1:1000	Servicebio, Wuhan, China	V2.0-20250930
BAX	1:1000	Proteintech Group, Wuhan, China	50599-2-Ig
Bcl-2	1:1000	Proteintech Group, Wuhan, China	26593-1-AP
Bcl-xL	1:1500	Proteintech Group, Wuhan, China	10783-1-AP
XIAP	1:1500	Proteintech Group, Wuhan, China	23453-1-AP
MMP3	1:1500	Proteintech Group, Wuhan, China	17873-1-AP
MMP9	1:1500	Proteintech Group, Wuhan, China	27306-1-AP
GAPDH	1:1000	Servicebio, Wuhan, China	V2.0-202506

As shown in Supporting Information Figure S1, pairwise comparisons of tissue samples metabolites across the BC, MC and PP-H groups revealed distinct metabolic profiles: in MC vs. BC, the model group exhibited marked upregulation of metabolites including Arg-Thr and downregulation of 4-Ethylphenol; in BC vs. PP-H, PP-H treatment significantly increased levels of Ubiquinone-1 while decreasing Acetylvanillic acid; and in MC vs. PP-H, the intervention reversed key metabolic dysregulation, elevating Ubiquinone-1 and reducing Hydroxysepiaepterin relative to the model group.

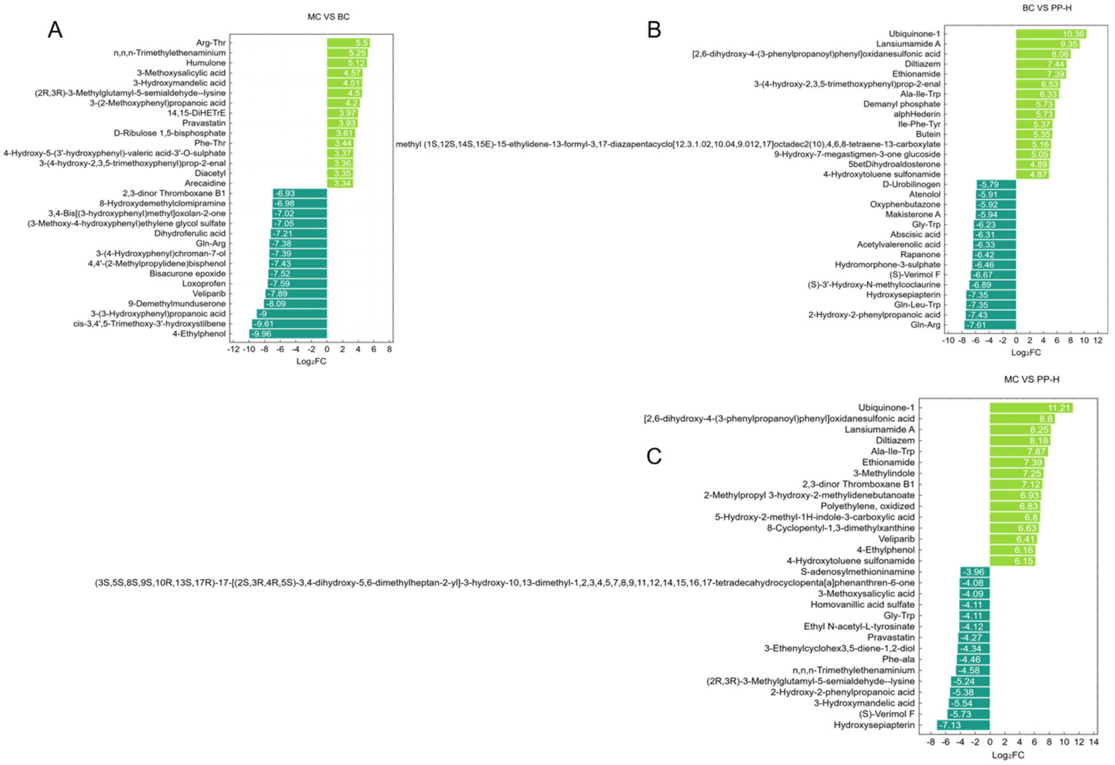

Supporting Information Figure S1. Differential metabolite Bar plots under three conditions.

Bar plots show the $\log_2$ fold change (Log_2FC) of significantly altered metabolites in three pairwise comparisons: A MC vs. BC, B BC vs. PP-H, and C MC vs. PP-H. Each bar represents a single metabolite, with green bars indicating upregulated metabolites and teal bars indicating downregulated metabolites. The horizontal axis represents the Log_2-transformed fold change in metabolite abundance between the two groups in each comparison.

Representative total ion chromatograms (TICs) from LC-MS analysis of PP samples are shown in Supporting Information Figure S2. The chromatographic separation achieved good resolution and signal intensity for metabolites in both the negative ionization mode (A) and positive ionization mode (B).All detailed data are presented in Supporting Information Excel Table 8.

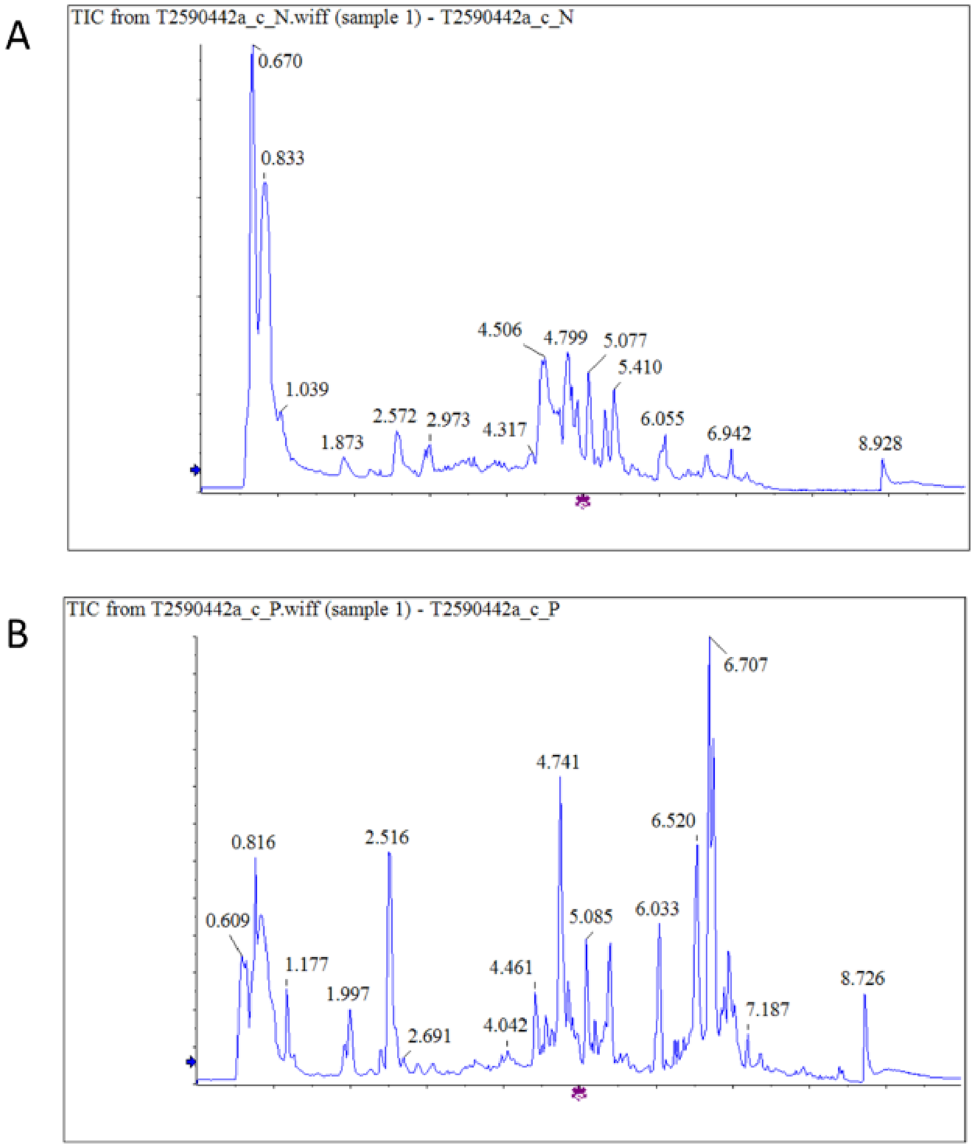

Supporting Information Figure S2.Representative LC-MS total ion chromatograms (TICs) of PP samples.

TICs were acquired in (A) negative ionization mode and (B) positive ionization mode. The horizontal axis represents retention time (min), and the vertical axis represents ion intensity.

Quality control (QC) samples, prepared by pooling equal aliquots of all study samples, were analyzed at intervals of every 10 test samples to monitor the reproducibility and stability of the LC-MS analysis. As shown in Supporting Information Figure S3, the overlaid total ion chromatograms (TICs) of QC samples (A: negative ionization mode; B: positive ionization mode) exhibited highly consistent peak shapes, retention times, and signal intensities across the entire analysis run. All detailed data are presented in Supporting Information Excel Table S7.

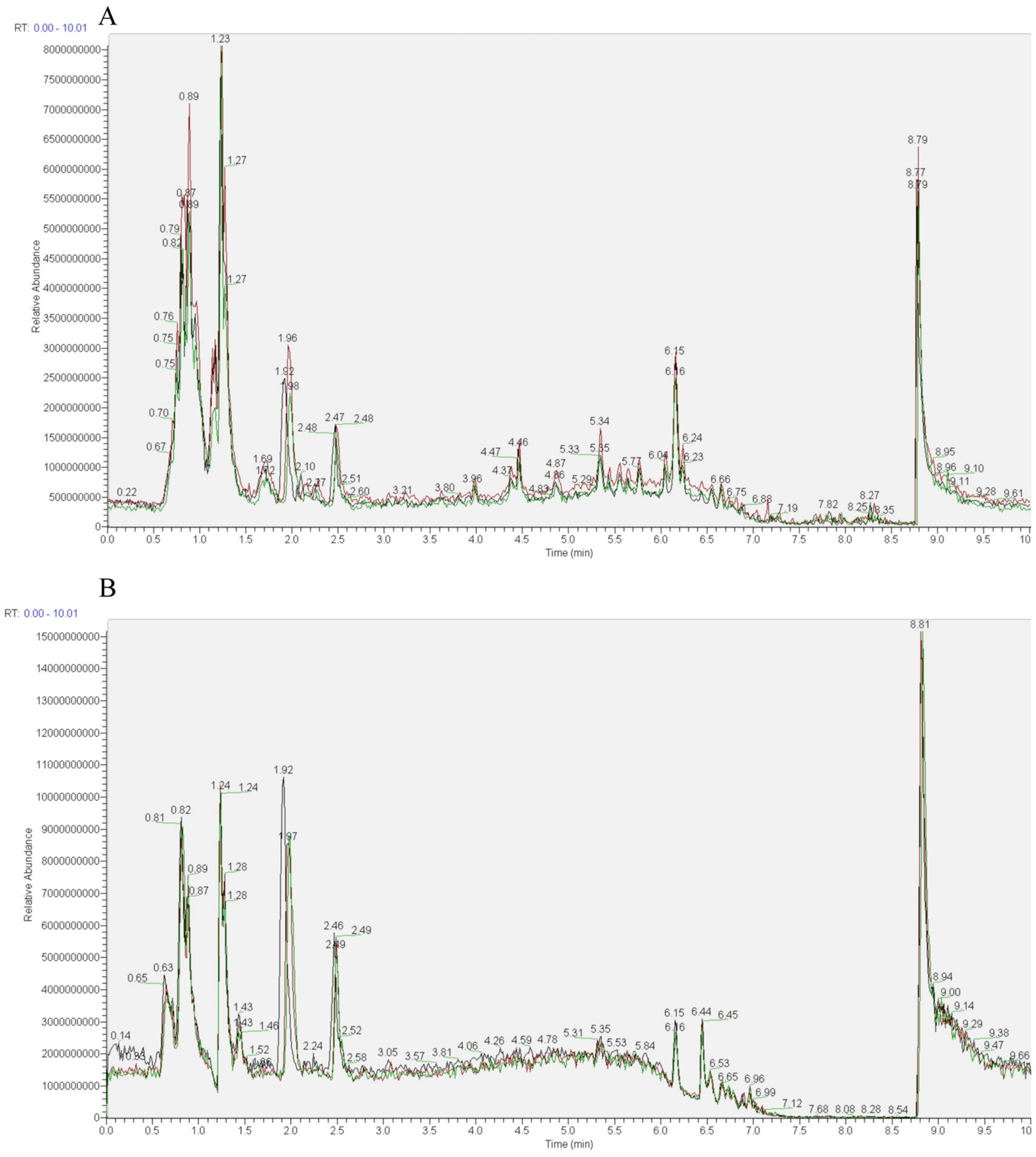

Supporting Information Figure S3 Overlaid total ion chromatograms (TICs) of quality control (QC) samples in negative and positive ionization modes.

QC samples were prepared by pooling equal volumes of all mouse tissue extracts and injected every 10 samples during the LC-MS analysis to assess system stability. (A) TICs acquired in negative ionization mode; (B) TICs acquired in positive

ionization mode. The horizontal axis represents retention time (min), and the vertical axis represents relative ion abundance. The high overlap of chromatographic profiles across QC samples indicates good analytical reproducibility.
